# Targeting the Interleukin-5 Pathway for Treatment of Eosinophilic Conditions Other than Asthma

**DOI:** 10.3389/fmed.2018.00049

**Published:** 2018-04-06

**Authors:** Florence Roufosse

**Affiliations:** Hôpital Erasme, Department of Internal Medicine, Université Libre de Bruxelles, Brussels, Belgium

**Keywords:** benralizumab, eosinophilic esophagitis, eosinophilic granulomatosis with polyangiitis, hypereosinophilic syndrome, interleukin-5, mepolizumab, nasal polyposis, reslizumab

## Abstract

Improved understanding of the contribution of eosinophils to various chronic inflammatory conditions, most notably allergic asthma, has encouraged development of monoclonal antibodies specifically targeting mediators and surface receptors involved in eosinophil expansion and activation. The pivotal role of interleukin-5 (IL-5) in eosinophil biology, its high specificity for this leukocyte subset, and its involvement in the majority of eosinophilic conditions make it a very enticing target for treatment of eosinophil-mediated disorders. Two types of antibodies have been developed to target eosinophils: antibodies against IL-5 (mepolizumab and reslizumab), and an antibody against the IL-5-receptor-alpha-chain (IL-5Rα) (benralizumab). Both types of antibodies prevent IL-5 from engaging its receptor and in addition, anti-IL-5Rα antibodies induce target-cell lysis. They have been shown to reduce circulating eosinophil counts rapidly in humans with various disorders. Herein, a brief overview of the role of IL-5 in eosinophil biology will be presented, followed by a description of the development and characteristics of antibodies targeting IL-5 or its receptor. Results of clinical trials evaluating the efficacy and safety of these new antibodies in diseases (other than eosinophilic asthma) with prominent tissue eosinophilia are reviewed, followed by safety considerations and potential future applications.

## Introduction

Improved understanding of the contribution of eosinophils to various chronic inflammatory conditions, most notably allergic asthma, has encouraged development of monoclonal antibodies specifically targeting mediators and surface receptors involved in eosinophil expansion and activation. Interleukin-5 (IL-5) is a key mediator acting at many levels of eosinophil biology. Importantly, this cytokine has a very narrow set of cellular targets as, in humans, only eosinophils, basophils and a subset of mast cells are known to express the IL-5Rα (CD125) chain. The pivotal role of IL-5 in eosinophil biology, as well as its high specificity for this leukocyte subset, makes it a very enticing target for treatment of eosinophil-mediated disorders.

Two types of antibodies have been developed to target eosinophils: antibodies against IL-5 (mepolizumab and reslizumab), and an antibody against the IL-5Rα chain (benralizumab). Anti-IL-5 antibodies bind to IL-5 and interfere with occupation of the IL-5R, whereas anti-IL-5Rα antibodies bind to the membrane-expressed receptor, and both inhibit signaling and induce cell lysis. Both types of antibodies have been shown to rapidly reduce eosinophil counts in peripheral blood in humans.

Herein, a brief overview of the role of IL-5 in eosinophil biology will be presented, followed by a description of the development and characteristics of antibodies targeting IL-5 or its receptor. Results of clinical trials evaluating the efficacy and safety of these new antibodies in diseases (other than eosinophilic asthma) with prominent tissue eosinophilia are reviewed, followed by safety considerations and potential future applications.

## Eosinophils and IL-5

Eosinophils derive from a myeloid multipotent progenitor in bone marrow, with GATA-1, PU-1, and c/EBP acting as key transcription factors for their differentiation (1). The importance of GATA-1 for eosinophil lineage commitment is reflected by the complete absence of eosinophils in mice following deletion of the high-affinity GATA binding site in the GATA-1 promoter [delta dblGATA eosinophil-deficient strain (2)]. Human eosinophil progenitors express CD34, CD38, and CD125 (IL-5Rα). They pursue their maturation and proliferation in response to transcription and growth factors, including most notably IL-5. As they mature, eosinophils produce eosinophil cationic protein (ECP), major basic protein, eosinophil peroxidase (EPO), and eosinophil-derived neurotoxin (EDN) that are stored in cytoplasmic granules. These cationic proteins account for eosinophil avidity for the acidic dye eosin. The specificity of EPO expression by eosinophils has been exploited to generate the transgenic PHIL eosinophil-less mouse strain, wherein the EPO promoter drives expression of diphtheria toxin A (3). Mature eosinophils also produce a multitude of cytokines, growth factors, chemokines, and lipid mediators.

Among the factors contributing to eosinophil maturation, IL-5 is the most specific. This cytokine functions as a homo-dimer and its receptor (IL-5R) is a hetero-dimer, with a ligand-binding alpha-subunit, and a non-ligand-binding signal transducing beta-subunit (4). The IL-5Rα chain is expressed only by eosinophils, basophils, and mast cells (with highest expression levels on the former) in humans. The common beta chain is also involved in intracellular signaling in response to IL-3 and granulocyte macrophage colony stimulating factor (GM-CSF), and in contrast to IL-5Rα, the ligand-binding receptor components for IL-3 and GM-CSF are shared by diverse cell types.

Interleukin-5 acts on eosinophils at multiple functional levels and time points during their life-span (5). Besides stimulating proliferation, differentiation and maturation of IL-5Rα-expressing eosinophil-committed progenitors in the marrow, IL-5 contributes to eosinophil egress from the marrow toward the intravascular compartment. When produced in tissues, this cytokine also synergizes with chemotactic factors such as eotaxin-1 (CCL11) to attract eosinophils (homing), and primes these cells for activation in response to various mediators. Finally, IL-5 prolongs eosinophil survival in concert with other anti-apoptotic factors. Thus, increased IL-5 production induces (hyper)eosinophilia (i.e., blood eosinophil count above 1.5 G/L and/or increased presence of eosinophils/eosinophil granule proteins in tissue), both by stimulating eosinophoiesis and by reducing peripheral apoptosis. Interestingly, however, IL-5 over-expression alone appears to be insufficient for induction of eosinophil-mediated damage, as evidenced in IL-5 transgenic mice that have marked eosinophilia in blood and certain tissues, without associated organ dysfunction (6). Furthermore, eosinophil maturation may occur independently of IL-5, as suggested by presence of eosinophils in blood and tissues in IL-5 knock-out mice (7). Indeed, these mice fail to mount hypereosinophilia in the setting of a Th2 immune response (8), but homeostatic eosinophils remain detectable. Recent studies confirm that homeostatic eosinophils have different response patterns and functions depending on their localization; resident eosinophils home to lungs and survive independently of IL-5, contrasting with those in adipose tissue (9). Furthermore, peripheral survival of mature eosinophils may be supported by IL-3 and/or GM-CSF through induction of bcl-xl expression (10). Thus, although IL-5 clearly plays a central role in eosinophil biology, it appears neither entirely necessary nor sufficient for certain eosinophil functions.

Most human diseases accompanied by hypereosinophilia are associated with increased IL-5 production (4). The most common source of IL-5 is “type 2” CD4^+^ helper T cells, either in the setting of an immune response to an environmental agent or pathogen (e.g., allergy and helminthiasis), or in the setting of T cell lymphoma (e.g., Sezary syndrome). In these conditions, IL-5 is often co-expressed with other cytokines including IL-4 and IL-13, resulting in associated increased vascular permeability, smooth muscle contractility, and IgE production. Other less common sources of IL-5 include transformed epithelial cells (e.g., cervical, colorectal, or non-small-cell lung cancer), and Reed-Sternberg cells in Hodgkin’s lymphoma. More recently, type 2 innate lymphoid cells have been shown to represent a source of IL-5 (11). These cells reside in the skin, lungs, and gastrointestinal tract and are activated in presence of alarmins (IL-33 and TSLP) and IL-25 (a.k.a. IL-17E). They contribute to eosinophilic inflammation in murine models of allergic asthma and are increased in sputum from patients with severe allergic asthma where they represent the predominant source of IL-5 (12).

Whatever the source of IL-5 may be, this cytokine selectively and broadly affects eosinophil biology in humans and is involved in the majority of diseases mediated by eosinophils. As such, IL-5 represents an appealing therapeutic target for hypereosinophilic conditions.

## Humanized Monoclonal Antibodies Targeting IL-5 and its Receptor

Three anti-IL-5 pathway therapies have been developed for clinical use (Table 1) (13, 14). Mepolizumab and reslizumab, both anti-IL-5 antibodies, bind to and neutralize soluble IL-5, thereby interfering with its ligation to IL-5Rα. Benralizumab is directed against the membrane-expressed IL-5Rα chain, and thereby recognizes (and binds) eosinophils directly. All three have been evaluated in asthmatic patients in large-scale clinical trials, from which most of the pharmacokinetic/dynamic data that follows has been derived. Studies in eosinophilic conditions other than asthma, which are the focus of this review, have been published only for mepolizumab and reslizumab so far. Abundant data is available about effects of treatment on blood eosinophilia, whereas only a few of the earlier small-scale studies have assessed bone marrow and tissue eosinophil responses.

**Table 1:** Antibodies targeting IL-5 and its receptor.

	Mepolizumab	Reslizumab	Benralizumab
Trade name	Nucala	Cinquair (USA), Cinquaero (EU)	Fasenra
Other names	SB-240563	SCH55700	MEDI-563
Company	GlaxoSmithKline	Teva	AstraZeneca/Medimmune
Regulatory approval	2015: FDA 4 Nov, EMA 2 Dec (asthma)	2016: FDA 23 Mar, EMA 16 Aug	2017: FDA Nov 14
Vial strength	100 mg	25 and 100 mg	30 mg (pre-filled syringe)
Route of administration	SC (formerly IV)	IV	SC
Dosing (approved in asthma)	100 mg SC/4 wks	3 mg/kg IV/4 wks	30 mg/4 wks (first 3 doses), then /8 wks
Dosing (other)	EGPA, HES: 300 mg	–	–
Type of Ig	IgG1, kappa humanized	IgG4, kappa humanized	IgG1, kappa humanized
Mechanism of action	Neutralizes free IL-5: prevents binding to IL-5Rα	Neutralizes free IL-5: prevents binding to IL-5Rα	Binds to IL-5Rα: interferes with binding of IL-5 and induces ADCC
Time to response, blood eos	1 day	1 day	1 day
Elimination half-life	SC: 16–26 days, IV: 28 days	24 days	15 days

*ADCC, antibody-dependent cellular cytotoxicity; EGPA, eosinophilic granulomatosis with polyangiitis; EMA, European Medicines Agency; eos, eosinophils; FDA, Federal Drug Administration; HES, hypereosinophilic syndrome; Ig, immunoglobulin; IL, interleukin; IL-5, interleukin-5; IV, intravenous; SC, subcutaneous; wk, week*.

### Anti-IL-5 Antibodies

*Mepolizumab* (Nucala^®^) is a fully humanized, IgG1-type antibody with high affinity and specificity for IL-5 (15). It has been administered intravenously (IV) and subcutaneously (SC) at various doses in a number of clinical trials conducted in eosinophil-mediated diseases and is currently approved (as first-in-class) for use as add-on therapy for patients with severe eosinophilic asthma, at the dose of 100 mg SC every 4 weeks. The route of elimination is unknown, but like other immunoglobulins, it is probably degraded by proteolytic enzymes. The dose need therefore not be adapted in patients with kidney or liver impairment. The bioavailability of SC mepolizumab is roughly 80%, with dose-proportional pharmacokinetics over a range of doses, and a median time to maximal concentration of 6–8 days post-dosing (compared with 30 min for IV) (16). When three consecutive doses are administered SC, the accumulation ratio is 1.7. The ratio between maximal mepolizumab concentrations reached in blood following monthly SC versus IV administration (when doses are normalized) is 42% after the first dose, and 54% after the third dose. The elimination half-life of SC mepolizumab is 16–22 days and slightly longer (28 days) for the IV route.

Pharmacodynamic and/or clinical studies have shown that the effect of mepolizumab on blood eosinophil levels is rapid and dose-dependent. Reduced eosinophilia is observed in blood already 24 h after administration (SC or IV), although levels continue to decline, with a peak reduction in asthma seen at 4 weeks (17). To determine the optimal dosing regimen in asthma, the extent of eosinophil depletion was quantified over a range of SC doses after three consecutive monthly injections; a 90% maximal reduction was achieved with a dose of 99 mg SC, whereas 11 mg only reached 50% of the maximal effect (15). In this line, posttreatment eosinophil levels were higher in asthmatic patients receiving 12.5 mg SC than in those treated with 125 mg SC, 250 mg SC, and 75 mg IV (16). The duration of the effect on eosinophils is also dose-dependent, in keeping with prolonged detection of mepolizumab in plasma as the dose increases (18). Depending on the dose, route of administration, and disease, the return of blood eosinophilia to baseline values varies. In patients with normal or marginally increased eosinophil counts, the effect of 100 mg SC or more lasts roughly 3 months (16). In patients with hypereosinophilic syndrome (HES, defined on the basis of blood eosinophilia of at least 1.5 G/L, i.e., 1,500/μL) in whom higher doses have been tested (750 mg IV) the duration of eosinophil depletion is variable, ranging from 3 to 37 weeks, with a median interval between infusions of 12.8 weeks (19, 20). This variability is likely related to the amount of endogenously produced IL-5 in this heterogeneous disease.

While eosinophil counts drop in mepolizumab-treated subjects, serum IL-5 levels have been shown to increase over time (16, 21). One group showed that most of the IL-5 detected during treatment is part of a complex, bound to an immunoglobulin (20) (most likely mepolizumab), and it has been hypothesized that the half-life of complexed IL-5 is prolonged. The biological significance and fate of these complexes remain unknown.

The effects of mepolizumab on bone marrow eosinophils have been examined in asthma and other eosinophilic disorders. One study with asthmatic patients showed a 70% decrease in mature eosinophil counts compared with placebo but no effects on CD34^+^ cells expressing the IL-5Rα receptor (early eosinophil progenitors) following mepolizumab administration, indicating that treatment leads to maturational arrest of the eosinophil lineage (22). Despite this observation, no major concerns have been raised with mepolizumab regarding enhanced eosinophil maturation once treatment is interrupted (see Safety of Therapeutic Antibodies Targeting IL-5 and Its Receptor).

Effects of mepolizumab on tissue eosinophils will be developed in detail below (see Clinical Trials Evaluating Antibodies That Target IL-5 or Its Receptor in Mucosal Eosinophilic Disorders besides Eosinophilic Asthma and Clinical Trials Evaluating Antibodies That Target IL-5 or Its Receptor in Systemic HESs). In asthmatic patients, bronchial mucosal eosinophils decrease by roughly 50% at maximal dosing (750 mg IV), regardless of the duration of treatment [similar findings after 3 (17) or 12 (23) monthly infusions].

*Reslizumab* (Cinquaero^®^ or Cinquair^®^), previously known as SCH55700, is a fully humanized, IgG4-type antibody with high affinity and specificity for IL-5 (24). It has been administered IV in clinical trials so far, and this route of administration has recently been approved in the USA and Europe for use as add-on maintenance therapy in adult patients with severe eosinophilic asthma. The SC route of administration is currently being assessed for treatment of asthma. Repeated dosing of reslizumab results in 1.5- to 2-fold accumulation relative to a single dose. The half-life of this antibody has been estimated at 24 days, and like mepolizumab, proteolytic degradation is the presumed mechanism of elimination.

The effects of reslizumab on blood eosinophil counts are dose-dependent, which probably explains the low (50%) response rate observed in an early study conducted in patients with HES treated with 1 mg/kg (25). Subsequent trials with higher dosing in patients with lower baseline eosinophil levels have confirmed the rapid and profound eosinophil-depleting effect, similar to mepolizumab. Effects on bone marrow eosinophilia have not been evaluated in asthma, but one study conducted on four patients with HES showed unchanged bone marrow cellularity and eosinophilia (25). Maturational arrest was not observed in aspirates from this small cohort of patients with markedly increased bone marrow eosinophilia and low-dose anti-IL-5 treatment.

Like mepolizumab, reslizumab increases the serum IL-5 level one month posttreatment in patients with HES; it remains unknown whether this represents free or complexed IL-5 (26). Culture-medium-containing serum from reslizumab-treated patients was shown to prolong eosinophil survival *in vitro*, leading investigators to hypothesize that anti-IL-5 may not only prolong half-life but also actually potentiate IL-5 activity in certain conditions.

### Anti-IL-5R Antibody

*Benralizumab* (Fasenra^®^) is a fully humanized, afucosylated IgG1-type anti-IL-5Rα antibody (27). This antibody binds to the IL-5Rα expressed by eosinophils and basophils, close to the site that binds IL-5, thereby hindering access of IL-5 to its receptor (and neutralizing its effects) and inducing target-cell depletion through natural killer cell-mediated antibody-dependent cellular cytotoxicity (ADCC). Benralizumab has just been approved by the FDA as add-on maintenance therapy for children (12 years and older) and adults with severe asthma and an eosinophilic phenotype. Afucosylation of this antibody results in marked enhancement of its affinity for the FcγRIIIa receptor on natural killer cells, thereby competing with non-specific endogenous IgGs, and making benralizumab a highly efficient cytotoxic antibody. Importantly, because of its mechanism of action, benralizumab can destroy IL-5Rα-expressing cells, regardless of their relative dependency on IL-5 or other mediators for their growth or survival. Moreover, ADCC is not significantly affected by the density of target antigen, so benralizumab is capable of destroying cells even if they display low-level expression of the IL-5Rα chain (13). Finally, the efficacy of benralizumab should theoretically not be decreased in presence of high-level endogenous IL-5 production, in contrast to anti-IL-5 antibodies.

Pharmacokinetic studies with benralizumab have shown a linear relationship between dosing and concentration. The volume of distribution exceeds that of the intravascular compartment, indicating potential binding to blood cells and/or access to the extravascular compartment (28). Benralizumab’s mean elimination half-life is roughly 18 days. The bioavailability of subcutaneous dosing is roughly 50%, and this route has pharmacokinetic/dynamic properties similar to IV dosing.

The depleting effect of benralizumab on peripheral blood eosinophils is particularly rapid and pronounced. At doses of 0.3 mg/kg IV and above, the maximal effect is observed at 24 h, at which time eosinophils are barely detectable (close to the limit of detection in healthy subjects and patients with asthma) (28, 29). Basophils also rapidly become undetectable with benralizumab, but this has been studied less extensively and the clinical relevance is unknown (30). One group investigated eosinophil biomarkers in benralizumab-treated asthmatic patients (3 monthly SC doses) to determine whether uncontrolled and potentially detrimental release of toxic eosinophil-derived mediators occurs at treatment initiation when eosinophils are destroyed (31). Serum levels of EDN and ECP were shown to decrease compared with baseline levels. Innocuity of eosinophil destruction by ADCC is further supported by the fact that none of the clinical trials in asthmatic patients have reported disease worsening at treatment initiation. Single-dosing studies have shown that eosinophil depletion is prolonged after administration of various doses of benralizumab, lasting at least 12 weeks for doses ranging from 0.3 to 3 mg/kg IV. At lower doses, the effect is less long-lasting (28). Because of the rapidity and duration of eosinophil depletion in response to benralizumab, it was tested in patients with acute asthma attacks presenting to the emergency department (32). Administration of a single dose of IV benralizumab (0.3 or 1 mg/kg) within 7 days in addition to standard of care reduced the frequency of subsequent exacerbations by 50%, and hospitalizations by 60%, over a period of 12 weeks compared with placebo.

Bone marrow eosinophils (precursors and mature cells) in asthmatic patients treated with a single IV (1 mg/kg) or 3 monthly SC (100 mg) doses of benralizumab are completely suppressed 4 weeks after dosing (30). Immunohistochemical staining of lung biopsies from asthmatic patients has shown that benralizumab stains more than 90% of eosinophils (33), indicating that effects on tissue eosinophilia could be dramatic, provided the antibody can access inflamed tissue. Bronchial biopsies obtained during a relatively small-scale placebo-controlled clinical trial before and after IV or SC benralizumab treatment showed that airway mucosal eosinophils decreased in 82% patients receiving active treatment, with a 96% median reduction after three consecutive SC doses (100 or 200 mg) (30). The effect was dose-dependent, with a less profound reduction following a single IV dose (1 mg/kg). The effects on tissue eosinophils with the dosing regimens used in the most recent clinical trials (30 mg SC at 4- or 8-week intervals) have not been assessed.

Similar to anti-IL-5 antibodies, treatment with benralizumab is followed by an increase in serum IL-5 levels as well as eotaxin-1 and -2 (but not eotaxin-3) (31). Presumed mechanisms include IL-5 accumulation in serum as a result of depletion of target receptors, and disruption of a negative autoregulatory loop whereby eosinophils inhibit IL-5 production.

### General Considerations

Overall, antibodies targeting IL-5 reduce blood eosinophil counts in a dose-dependent manner, with dramatic reductions observed at sufficient dosing. The mechanisms of eosinophil depletion have not been fully elucidated. Maturational arrest has been demonstrated in the bone marrow of mepolizumab-treated asthmatics. The rapidity of the drop in blood eosinophil counts suggests additional peripheral mechanisms that may include apoptosis through cytokine deprivation. The effects of these antibodies on tissue eosinophils are less pronounced, often closer to a twofold reduction. This may be explained by restricted access of these antibodies to tissues, the contribution of mediators other than IL-5 to eosinophil survival in tissue, and/or shedding of the IL-5Rα chain by activated tissue-infiltrating eosinophils (34). Whether residual tissue eosinophilia accounts for some of the disappointing clinical responses observed with anti-IL-5 treatment remains unknown (35).

Anti-IL-5R antibodies have been shown to deplete tissue eosinophils more profoundly in asthmatic subjects. Future clinical trials with anti-IL-5R may finally clarify the true role played by eosinophils in organ damage and dysfunction in other eosinophilic conditions, such as eosinophilic esophagitis (EoE). A potential limitation to efficacy of benralizumab in disorders with marked expansion of activated eosinophils may be enhanced membrane cleavage of IL-5Rα with shedding of its soluble form (sIL-5Rα) and/or alternative splicing of IL-5Rα mRNA (36). Indeed, serum levels of sIL-5Rα have been shown to rise with increasing eosinophilia, while membrane expression decreases, in subjects with hypereosinophilia. The soluble form may intercept benralizumab before it can access target cells. The results of an ongoing placebo-controlled clinical trial evaluating efficacy of benralizumab in patients with HES, who commonly have increased serum sIL-5Rα (36), should shed some light on this question.

## A Brief Historical Perspective on the Development of IL-5 Targeted Therapy for Human Diseases

Well before the development of therapeutic antibodies targeting the IL-5 pathway, numerous observations in humans and mice established the eosinophil as a key player in the pathogenesis of allergic airway disease. In asthmatic humans, blood and airway eosinophil counts were shown to increase with clinical severity, and histopathological studies showed that bronchial epithelial shedding was associated with close proximity of degranulated eosinophils (37). Furthermore, in murine models of experimental asthma, either genetic manipulation of IL-5 expression or pretreatment of mice with anti-IL-5 antibodies abolished blood and airway eosinophilia, prevented development of airway damage, and reduced airway hyperreactivity, confirming the key role both of eosinophils and IL-5 in this disease (8, 38).

Two companies (GlaxoSmithKline and Schering-Plough) developed anti-IL-5 antibodies (mepolizumab and SCH55700/reslizumab, respectively) at the same period, and a third company subsequently developed an anti-IL-5Rα antibody (AstraZeneca-Medimmune, benralizumab), with the intention of improving asthma control and reducing the need for poorly tolerated anti-inflammatory agents such as oral corticosteroids (OCS). Establishing the efficacy of eosinophil-depleting antibodies in asthma turned out to be challenging, with a particularly long interval between the first clinical trial (published in 2000) and regulatory approval of the first anti-IL-5 antibody for severe eosinophilic asthma in 2015. Indeed, initial trials with anti-IL-5 antibodies enrolled “all-comer” asthmatic patients regardless of disease severity, phenotype or endotype (13, 39), and although blood and sputum eosinophils decreased significantly, no improvement in lung function was observed. It took several years to identify the ideal candidates for IL-5 targeted therapy, based on a better understanding of asthma heterogeneity.

In the meantime, the two companies producing anti-IL-5 approached an entirely different medical community to seek validation of the concept that neutralizing IL-5 results in eosinophil depletion, and control of eosinophil-mediated disease. HESs compose a heterogeneous group of diseases characterized by a marked increase in blood and/or tissue eosinophils, associated with organ dysfunction and damage for which no cause other than eosinophil toxicity can be detected. Mepolizumab was administered to a handful of patients with HES in two short mono-centric open-label studies (40, 41), the clinical results of which were so encouraging that orphan drug status was granted, and an international placebo-controlled double-blind randomized clinical trial was undertaken to assess efficacy in this rare disease in 2004 (42) (see Clinical Trials Evaluating Antibodies That Target IL-5 or Its Receptor in Systemic HESs). Although the results of this trial confirmed that mepolizumab was an effective CS-sparing agent for patients with HES, regulatory authorities judged that the trial design was flawed and requested additional data supporting use of anti-IL-5 in this indication. Indeed, (1) physicians were not blinded to eosinophil counts, and were therefore practically speaking not blinded to treatment, given the clear-cut eosinophil-depleting effect of mepolizumab, (2) disease was controlled with maintenance OCS treatment at baseline, and the fact that disease control was maintained despite significant OCS tapering in the active treatment arm was not considered a valid surrogate for a clinical response to mepolizumab, and (3) patients in the placebo arm had significantly shorter exposure to drug than patients in the active treatment arm, because the trial design permitted early withdrawal and open-label access to mepolizumab after the first two study-drug infusions. GlaxoSmithKline withdrew its marketing authorization application for mepolizumab in HES in 2009, and a long effort toward designing a trial that would address regulatory concerns began.

Notwithstanding, proof of concept was clearly achieved in HES, and with improved characterization of asthma phenotypes, a more accurate picture of the type of patient most likely to benefit from therapeutic eosinophil-targeting emerged. Two pilot studies were undertaken to evaluate mepolizumab versus placebo in patients with severe asthma and persistent eosinophilic inflammation (a factor known to be associated with asthma exacerbations) despite high-dose inhaled CS use (23, 43). As expected, these studies showed rapid normalization of blood and sputum eosinophil counts in the active treatment arm, but more importantly, a significant reduction in the exacerbation rate in mepolizumab-treated compared with placebo-treated patients was observed. Patients requiring long-term OCS treatment to maintain disease control before inclusion were better able to lower their OCS dose in the active-treatment arm. The two landmark studies were published back-to-back in 2009 and were followed by a series of large-scale placebo-controlled trials that consistently confirmed the added value of anti-IL-5(R) treatment in severe eosinophilic asthma, with decreased exacerbation rates relative to placebo, improved ability to taper OCS, increased forced expiratory volume, all reflected by better clinical asthma scores. The trials involved in establishing the efficacy of IL-5 pathway targeting in asthma, leading to regulatory approval, have recently been reviewed (44).

As for patients with HES, more than 10 years after the first large-scale clinical trial, mepolizumab is now being tested in a randomized placebo-controlled trial that will be pivotal in seeking regulatory approval for this rare disorder. The trial has been designed to truly assess the clinical efficacy of anti-IL-5 and should guarantee double-blinding. Indeed, the primary endpoint is related to disease flares, and physicians will be blinded to eosinophil counts.

The long story of anti-IL-5(R) development illustrates nicely how rare diseases, with homogenous (and occasionally well delineated) pathogenic mechanisms, represent powerful tools to establish proof of concept for the development of highly targeted therapeutic compounds (45). Thus, patients with rare diseases are finally offered opportunities to access efficacious treatment through clinical trial participation, followed by open-label long-term access programs and regulatory approval. In turn, biomarker data collected during these studies can be used to improve selection of patients for large-scale clinical trial implementation in the setting of more common, but also more heterogeneous, illnesses (46).

## Clinical Trials Evaluating Antibodies That Target IL-5 or its Receptor in Mucosal Eosinophilic Disorders Besides Eosinophilic Asthma

Eosinophilic asthma is one of several disorders wherein eosinophils participate massively to inflammatory infiltrates in mucosal tissue; these include EoE and chronic rhinosinusitis, especially in presence of nasal polyps (CRSwNP). Like asthma, blood eosinophilia is often mild (if present) in these disorders, and pathogenic mechanisms likely include allergic sensitization, and numerous cell types and mediators beyond eosinophils and IL-5. Targeting IL-5 in eosinophilic asthma has nonetheless been shown to improve certain disease components and reduce the need for OCS. Effects of anti-IL-5 antibodies in EoE and CRSwNP have been evaluated in several clinical trials (Table 2).

**Table 2:** Clinical trials evaluating anti-IL-5 antibodies in mucosal eosinophilic disorders other than asthma.

Reference Drug Clinicaltrials.gov ID	Study design Dose Route # Injections Interval	Patients			Response to *active* treatment^a^				
		Age (yrs)	Nbr	Baseline disease characteristics	Primary EP	Blood EOS	Tissue EOS	Clinical (symptoms/signs)	Other endpoints/findings
**Eosinophilic Esophagitis**									

Stein et al. (47) MEPO NCT00266565	Open label 10 mg/kg (max 750) IV 3× 4 wks	18–41	4	Sympt >9 yrs Strictures (3/4) CS resistant (2/4) EsoEOS > 24/hpf	–	Mean sixfold decrease (444 to 69.5/mm^3^) Still low 12 wks after last infusion	Mean ninefold decrease Resolution of eos microabsc (2/2)	Variable symptom improvement (4/4): reduced dysphagia, vomiting, food impactions, pain, and diet advancement	Endoscopy improved in 3/4 (persistent thickening and furrowing in 1)

Straumann et al. (48) MEPO NCT00274703	RDB PC 750 (2×) then 1,500 (2×) mg IV 4× d0, d7, wk5, wk9	>18, mean 33	11	Peak EsoEOS > 20/hpf Mean peak eos at BL 200/hpf At inclusion: dysphagia present, off all EoE Tx History: poor response to topical/oral CS, food impactions	Peak esoEOS < 5/hpf 4 wks after 2× 750 mg: 0/5 patients	Decreased at wk1, and 12 wks after last infusion (up to 10-fold) Return to BL 34 wks after last infusion	No patients below 15 eos/hpf 65–72% reduction peak/mean eos counts at wks 4 + 13 (twofold to threefold)	No significant difference btw MEPO and PLAC Days with dysphagia decreased by 20/30% (wks 9–13/13–17) with MEPO and by 20/18% with PLAC	Endoscopy: 3/5 showed improvement with MEPO versus 2/6 with PLAC
									No significant differences in endoscopic response btw groups

Assa’ad et al. (18) MEPO NCT00358449	RDB (no PLAC) 0.55, 2.5, and 10 mg/kg (3 arms) IV 3× 4 wks	2–17, mean 10.4	59	Peak EsoEOS ≥ 20/hpf At inclusion: 19% asymptomatic, no info on ongoing EoE Tx History: poor response/tolerance to prior Tx	Peak EsoEOS < 5/hpf 4 wks after 3rd infusion:8.8% patients (no dose response)	Decreased at d1, wk12 More rapid recurrence in 0.55 mg/kg group No rebound	4 wks after 3rd infusion: – Peak EsoEOS fell to <20/hpf in 32% subjects (all 3 doses) – Peak/Mean EsoEOS decreased threefold/fourfold	No significant changes in symptoms Low symptom scores at BL; study not powered to detect changes	Endoscopy: reduced erythema, vertical lines, furrows in PR (EsoEOS < 20/hpf), and CR (EsoEOS < 5/hpf)
									*Predictor reduction mean eos count: higher epithelial BL eos count*
							16 wks after 3rd infusion: Peak/Mean EsoEOS remained below BL; lowest in 10 mg/kg group		

Spergel et al. (49) RESLI NCT00538434	RDB PC 1, 2, and 3 mg/kg (4 arms) IV 4× 4 wks	5–18	226	Peak EsoEOS ≥ 24/hpf Median peak EsoEOS 80/hpf At inclusion: ≥1 active symptom (moderate severity or worse), no topical or oral CS, diet (maintained)	1. % Change peak EsoEOS count: twofold reduction 2. Physicians EoE GAS: no significant difference	Not reported	Few patients had <5 EsoEOS/hpf at end of study: 2 PLAC, 8 RESLI (1 and 2 mg/kg arms) (all CR had peak BL EsoEOS < 60/hpf) Fold reduction in peak EsoEOS slightly higher in the RESLI 3 mg/kg arm	Symptomatic improvement in all groups (PLAC and RESLI): physician GAS, patient predominant EoE symptom score	Endoscopy not reported
									Followed by open-label extension study NCT00635089 evaluating long-term safety and efficacy (no published results)

**Chronic rhinosinusitis with nasal polyposis**									

Gevaert et al. (50) RESLI	Phase I, PoC, RDB PC 1 and 3 mg/kg (3 arms) IV 1×	18–63	24	Severe CRSwNP, with bilateral grade 3/4 polyps, or recurrence after surgery At inclusion: no local Tx or oral CS	Nasal polyp score at wk12: significant decrease only in the RESLI1 mg/kg arm	Reduced at d1, wk8 Back to BL at wk12 Rebound in 10/16 patients, at wk24 (1 mg/kg) and 32 (3 mg/kg)	Not assessed	No improvement in symptom scores or nasal PIF No rebound after treatment	50% patients had ≥1-point reduction in NP score (up to wk4)

Gevaert et al. (51) MEPO	RDB PC PLAC *n* = 10, MEPO *n* = 20 750 mg IV 2× 4 wks	Mean 48	30	Severe CRSwNP with grade 3/4 polyps, or recurrence after surgery, refractory to topical CS At inclusion: no local Tx or oral CS	Change from BL in total NP score 4 wks after 2nd infusion: −1.30 (PLAC 0.00, *P* = 0.028)	Reduced at wk1 (NS), wk4, wk8 Reduced to <200 in all subjects at wk8 No rebound	Not assessed	No significant improvement in symptom scores or nasal PIF *Improved olfaction, postnasal drip, congestion (not rhinorrhea) at wk8 (NS) Increased nasal PIF—(NS)* *Improved olfaction very durable (11 mo) when present*	60% patients had ≥1-point reduction in NP score at wk8 (versus 10% PLAC)
									> 50% had improved CT findings (versus <20% PLAC)
									In responders (≥1-point reduction NP score), effect maintained 36 wks after last infusion

Bachert et al. (52) MEPO NCT01362244	RDB PC 750 mg IV 6× 4 wks	18–70	105	Severe NP requiring surgery (nasal polyp score ≥3 + VAS symptom score >7) At inclusion: topical CS (standardized dose) History: refractory to SOC Tx, ≥1 prior surgery	Need for surgery 4 wks after 6th infusion: 30% reduction (PLAC 10%, *P* = 0.006)	Reduced from GM 500 to 80/mm^3^ after 1 wk, 50/mm^3^ 4 wks after 6th infusion	Not assessed	Improvement in VAS score for NP severity (-1.8 after 6 infusions) Improvement in VAS score for rhinorrhea and nasal blockage (delay 4 wks), mucus and anosmia (delay 8 wks) Improved SNOT-22 score after 6th infusion Nasal PIF improved after 6th infusion	Sixfold increased probability of having improved NP score after the 2nd infusion
									50% patients had ≥1-point reduction NP score (versus 27% PLAC)
									No association btw baseline eos counts and reduction of NP score

*^a^For placebo-controlled trials, the reported findings concern statistically significant (unless mentioned otherwise) differences observed between the active treatment arm and the placebo arm; the response rate to active treatment arm is reported first, followed by response to placebo. Several non-significant differences judged worth underlining are mentioned as well*.

*BL, baseline; btw, between; CR, complete response(ders); CRSwNP, chronic rhinosinusitis with nasal polyposis; CS, corticosteroid; d, day; EoE, eosinophilic esophagitis; eos, eosinophil; EP, endpoint; EsoEOS, esophageal epithelial eosinophils; GAS, global assessment score; GM, geometric mean; hpf, high-power field; IL-5, interleukin-5; IV, intravenous; MEPO, mepolizumab; mo, month; Nbr, number; NP, nasal polyposis; NS, non-significant; PIF, peak inspiratory flow; PLAC, placebo; PoC, proof of concept; PR, partial responders; RDB PC, randomized double-blind placebo-controlled; RESLI, reslizumab; SC, subcutaneous; SNOT, sinonasal outcome test; SOC, standard of care; Tx, treatment; VAS, visual analogy scale; wk, week; yr, year*.

### Eosinophilic Esophagitis

Eosinophilic esophagitis is a Th2-mediated inflammatory disease involving the esophagus, characterized by symptoms of esophageal dysfunction, increased eosinophil counts in esophageal biopsies (>15/high-power field) with epithelial hyperplasia, and lack of response to treatment directed against gastro-esophageal reflux disease (53). Frequent sensitization to food allergens has provided rationale for treatment strategies based on elimination of the most common food allergens (six-food elimination diet), and often impracticable amino acid-based diets. Other approaches include swallowing inhaled CS, and OCS therapy, with variable efficacy and significant long-term toxicity. In severe disease, endoluminal dilatation, enteral feeding, or parenteral nutrition may be required. EoE is therefore potentially a profoundly debilitating disease for which classical therapeutic options are difficult to adhere to and/or tolerate.

Animal models and translational research on large patient cohorts have led to a better understanding of pathogenesis, and to elaboration of targeted strategies. IL-5 is a key mediator in murine models of allergen- and IL-13-induced EoE, as evidenced by abolished esophageal eosinophilia and reduced remodeling in IL-5-deficient or anti-IL-5-treated mice (54, 55).

Anti-IL-5 treatment was first assessed in EoE in a single adult patient with refractory disease, in the setting of a small open-label study evaluating efficacy of monthly mepolizumab infusions (40). Biological, clinical (dysphagia and vomiting), and histopathological improvement of disease was observed, encouraging the same group to evaluate three additional patients with long-standing symptomatic EoE (47). This pilot study confirmed that 3 monthly infusions of mepolizumab reduced clinical manifestations, increased quality of life scores, and improved endoscopic appearance (narrowing and strictures) although esophageal thickening and furrowing persisted in one patient. A significant reduction of esophageal eosinophilia was observed in all four subjects (mean ninefold), but peak residual counts remained above 20/hpf. The clinical findings were deemed sufficiently promising to design several randomized double-blind trials with anti-IL-5 antibodies in adults and children with EoE.

One group evaluated mepolizumab (750 mg) versus placebo in 11 adults with treatment-refractory symptomatic EoE, using a very stringent primary endpoint: peak esophageal eosinophilia <5/hpf after 2 weekly infusions of study-drug (48). Because none of the patients reached this endpoint, two additional infusions of high-dose mepolizumab (1,500 mg) or placebo were administered at 4-week intervals. Biopsies showed a roughly threefold (65%) reduction in peak/mean eosinophil counts only in mepolizumab-treated patients. Findings were similar after the second and fourth infusions, indicating that the maximal histological (eosinophilic) response to 750 mg IV mepolizumab is achieved rapidly, with no further dose–response. Endoscopic appearance of the esophagus was not significantly improved by active treatment, and clinical benefit was marginal (both treatment groups experienced a reduction in the proportion of days with dysphagia, that was slightly more substantial at later time points in the active treatment arm).

Effects of anti-IL-5 on pediatric EoE were assessed in two large-scale multicenter studies published shortly thereafter, one with mepolizumab (18), the other with reslizumab (49). Three doses of each drug were tested, but only the reslizumab trial included a placebo arm. Histological findings were comparable to adults: although very few children experienced complete remission (i.e., peak eos <5/hpf), a partial response, with an overall twofold to threefold reduction in peak eosinophilia, was observed in many cases. In the mepolizumab trial, most eosinophilic microabscesses disappeared and had not recurred at the long-term follow-up visit 16 weeks after the third dose, although tissue eosinophilia was increasing. Furthermore, treatment responders also displayed endoscopic regression of erythema, vertical lines and furrows. Neither of the studies showed substantial clinical benefit with anti-IL-5: the mepolizumab study enrolled patients who were largely symptom-free at enrollment and was therefore not powered to detect significant improvements, and in the reslizumab trial, symptomatic improvement was observed in all groups including the placebo arm.

Overall, clinical trials in EoE strongly support a role for IL-5 in eosinophil accumulation in the esophageal epithelium, as most patients receiving active treatment (mepolizumab and reslizumab) experience a roughly 50–60% reduction in esophageal eosinophilia. However, only a minority of patients have a complete histological response (peak eosinophilia <5/hpf), with peak eosinophilia often remaining above the 15/hpf threshold defining EoE. The maximal effect of anti-IL-5 on esophageal eosinophilia appears to reach a plateau within weeks, at which point no further improvements can be achieved by increasing the dosing regimen (48). Furthermore, the effect of treatment on symptoms is inconsistent.

There are a number of potential explanations for the disappointing clinical response to anti-IL-5 in these trials. First, the residual tissue eosinophilia observed in the majority of treated patients may perpetuate disease activity and symptoms. Unfortunately, no data is available on individual clinical responses in the few patients who did normalize their esophageal eosinophil counts with anti-IL-5. Second, subepithelial fibrosis (remodeling) may contribute to symptom burden and be less amenable to reversal with therapy, especially in adult patients who often have long-standing disease. One group has shown, however, that short-term treatment with mepolizumab led to decreased esophageal expression of tenascin C and TGFβ, both of which are involved in remodeling (48). Clinical trials in EoE conducted so far may have been too short (only 3–4 monthly doses) for reversal of fibrosis and its functional consequences. Perhaps more prolonged reduction of eosinophilic inflammation is required to translate clinically into symptomatic improvement. One group recently reported their experience with a small cohort of children treated for up to 9 years with reslizumab (56) [enrolled in a open-label extension study then a compassionate use program, following participation in a randomized trial (49)], showing clear-cut symptom improvement and absence of disease progression despite a relatively unrestricted diet and no topical CS during this prolonged observation period. Third, cell types other than eosinophils and mediators other than IL-5 may contribute to EoE symptomatology. Indeed, the relationship between esophageal eosinophilia and symptoms is poor across various clinical conditions and therapeutic strategies (49, 57). Pathogenic mechanisms of EoE also involve alarmins (TSLP), IL-13 and its transcriptional targets, epithelial barrier dysfunction, and mast cells (58). One study on pediatric EoE for example has shown that in patients whose mast cell counts decrease most with mepolizumab treatment, baseline mast cell (but not eosinophil) counts are correlated with severity of pain (59), suggesting that mast cells may specifically contribute to this clinical manifestation.

Future trials with eosinophil-targeting compounds that consistently induce more profound tissue eosinophil depletion, such as benralizumab, should provide insight on whether eosinophils are central players in EoE symptoms once and for all. Should this prove not to be the case, combined therapy may yield better results, with topical CS potentially enhancing effects of antibodies targeting eosinophils and key cytokines such as IL-13 and/or chemokines.

### Chronic Rhinosinusitis with Nasal Polyposis

Among patients with chronic rhinosinusitis, those with nasal polyposis (CRSwNP) experience a particularly debilitating disease course, with refractory disease that recurs after surgery and a frequent association with severe asthma (60). Clinical manifestations impact quality of life significantly, with nasal obstruction, anosmia, nasal discharge, and headache. Treatment generally associates topical CS, and repeated courses of antibiotics and OCS to alleviate symptom exacerbation. This condition is associated with a Th2-type immune response in Caucasians, associating eosinophilic inflammation and elevated IL-5 levels in nasal secretions and tissue. When cultured nasal polyps are subjected to various *in vitro* treatments, only antibodies directed against IL-5 (but not IL-3 or GM-CSF) induce eosinophil apoptosis and deplete tissue eosinophils (61). These observations have provided rationale for clinical trials evaluating efficacy of antibodies targeting the IL-5 pathway in CRSwNP (Table 2). This disease offers the advantage of easy non-invasive access to tissue (polyps) and secretions for assessment of treatment effects on eosinophilia and soluble biomarkers.

An early phase 1 placebo-controlled clinical trial assessed the safety and efficacy of a single dose of reslizumab (1 or 3 mg/kg) in patients with severe (grade 3 and 4) bilateral nasal polyps (50). Although blood eosinophilia decreased in reslizumab-treated patients, the nasal polyp score decreased in only half of these subjects, and no significant improvements were noted in symptom scores or peak nasal flow. Patients whose nasal polyp score decreased had significantly higher baseline IL-5 levels in nasal secretions than non-responders. In fact, an IL-5 level above 40 pg/mL was the only predictive marker for a clinical response to reslizumab. Rebound blood eosinophilia was observed in two-thirds of anti-IL-5-treated patients, but posttreatment nasal polyp scores did not worsen.

The same group subsequently assessed the efficacy of 2 monthly mepolizumab infusions (750 mg) in patients with CRSwNP in a double-blind placebo-controlled study with a prolonged observation period (48 weeks) (51). An early and durable reduction in the endoscopic nasal polyp score was observed in 60% of mepolizumab-treated patients versus 10% in the placebo arm. The extent of the improvement was more pronounced than that observed in clinical trials with topical CS. Sinus CT findings were also significantly better after active treatment, and a trend toward symptom improvement was observed. Interestingly, the increased sense of smell experienced by certain patients was prolonged, contrasting with other symptoms (congestion and postnasal drip) that recurred more rapidly. Because patients were not allowed to use rescue intranasal therapy for the first 2 months, early withdrawals were numerous. The facts that the time to dropout was significantly longer in the mepolizumab-treated group, and that more placebo-treated patients required OCS therapy or surgery after withdrawal, provide additional indirect support for efficacy of mepolizumab in this disease. Rebound eosinophilia was not observed in this trial, and in contrast to the prior study with reslizumab, the level of IL-5 in baseline nasal secretions was not predictive of endoscopic improvement.

More recently, a large-scale trial focusing on the clinical outcome of patients with severe CRSwNP has shown a beneficial effect of mepolizumab on the requirement for surgery (52). In this double-blind placebo-controlled study, patients fulfilling endoscopic and symptomatic criteria for surgery were randomized to receive 6 monthly infusions of 750 mg mepolizumab or placebo. A higher proportion of mepolizumab- than placebo-treated patients no longer required surgery 4 weeks after the sixth infusion (30 versus 10%, respectively). Symptom scores and the quality of life SNOT-22 score improved. Interestingly, the time-to-improvement of individual symptoms varied; rhinorrhea and nasal obstruction regressed more rapidly (4 weeks) than anosmia (8 weeks). Among patients receiving active treatment, two achieved the primary endpoint only after the sixth infusion, suggesting that longer treatment duration may further increase the beneficial effect on requirement for surgery. Effects persisted well after treatment cessation, but too few patients entered the posttreatment extension phase for accurate assessment.

Chronic rhinosinusitis with nasal polyposis may be observed in patients with asthma and eosinophilic granulomatosis with polyangiitis (EGPA), and as such, has also been taken into consideration in clinical trials testing efficacy of anti-IL-5 in these disorders. Interestingly, one trial evaluating efficacy of reslizumab in severe eosinophilic asthma has shown that only patients with associated nasal polyposis experience significant clinical improvement (62) (asthma control questionnaires), suggesting that eosinophils may contribute more to the symptomatic burden and/or pathogenesis of asthma in this patient sub-group (63). In the recent study evaluating mepolizumab in EGPA (see Clinical Trials Evaluating Antibodies That Target IL-5 or Its Receptor in Systemic HESs), 94% of enrolled subjects had sinonasal abnormalities (64). Active treatment induced a significant reduction in the SNOT-22 score compared with placebo, and in the occurrence of EGPA relapses involving worsening of sinonasal symptoms, indicating that CRS associated with more complex systemic disorders may also benefit from IL-5 targeted therapy.

In summary, anti-IL-5 has the capacity to reduce the size and number of nasal polyps in patients with CRSwNP, and to reduce the need for surgery. Although this treatment option may seem unreasonably expensive for a disease that does not target vital organs, other financial considerations like the need for repeated surgery and decreased work productivity should be taken into account. IL-5 targeting appears to have prolonged effects and could be administered intermittently to forestall surgery. Costs may be further limited by dose reduction, an option that is currently being evaluated in a trial with monthly subcutaneous injections of 100 mg mepolizumab.

## Clinical Trials Evaluating Antibodies That Target IL-5 or its Receptor in Systemic HESs

Hypereosinophilic syndromes are rare and often debilitating chronic inflammatory disorders characterized by blood and tissue eosinophilia, with associated eosinophil-mediated organ damage and/or dysfunction. These disorders are currently classified on the basis of underlying molecular and immunological defects and the spectrum of target organ damage (65, 66) [see Kahn in this research topic (67)]. Although the mechanisms resulting in eosinophil expansion remain unknown in the majority of patients (“idiopathic” HES variant), the role played by eosinophils in tissue damage is undeniable and targeting the IL-5 pathway makes sense. The one disease variant for which IL-5 targeting should not be considered an option is chronic eosinophilic leukemia (CEL) with well-documented underlying cytogenetic rearrangements, most commonly the FIP1L1/PDGFRA (F/P) fusion gene (68). Even though mepolizumab did actually reduce blood eosinophilia in one patient with F/P^+^ CEL (19), such patients respond exquisitely well to low-dose imatinib mesylate that selectively targets the molecular default that drives disease and may even offer the prospect of cure. This section will focus on studies evaluating efficacy of IL-5-targeted treatment in systemic HES (namely, idiopathic and lymphocytic variants) and EGPA (Table 3). Only mepolizumab has been assessed repeatedly and in large-scale trials so far.

**Table 3:** Clinical trials evaluating anti-IL-5 antibodies in systemic hypereosinophilic disorders.

Reference Drug Clinicaltrials.gov ID	Study design Dose Route # Injections Interval	Patients			Response to *active* treatment^a^				
		Age (yrs)	Nbr	Baseline Disease Characteristics	Primary EP	Blood EOS	Tissue EOS	Clinical (symptoms/signs)	Other endpoints/findings
**Hypereosinophilic Syndrome**									

Plotz et al. (40) MEPO	Open-label 750 mg IV Variable (2, 8, and 10) d1, wk2, then monthly	60, 62, and 82	3	OCS-resistant eosinophilic dermatitis Patient 2: +fever and abd pain BL blood EOS > 1 G/L	–	Reduced at d1 post-Tx	Disappearance of skin EOS 1 wk after 2nd infusion	Resolution of pruritus and skin lesions (delay 3 d to 3 wks)	Prolonged 17-month remission after 2 infusions in 1 patient
						4- to 37-fold reduction 1 wk after 2nd infusion			
							Twofold to eightfold reduction skin ECP^+^ cells		

Garrett et al. (41) MEPO	Open-label 10 mg/kg (max 750) IV 3× 4 wks	40, 48, and 55	3^b^	Systemic F/P^−^ HES Run-in period: reduction of HES Tx (EOS increase twofold or >0.75 G/L)	–	Reduced in all 3 patients from wk2 to 12 wks after 3rd dose	Not assessed	Improved in all patients: skin, nasal congestion Improved FEV1, polyposis, and exercise tolerance	

Klion et al. (25) RESLI NCT00017862	Open-label 1 mg/kg IV Single dose; +5 doses if response 4 wks	32–52	4	Systemic HES1 F/P^+^ patient BL blood EOS > 2.5 G/L despite maintenance treatment	–	Rapid reduction in 3 patients Duration: 7 d in 1 case, >30 d in 2 cases	Not assessed	Improved in 2 patients: rash, mucosal ulcerations, angioedema, and arthromyalgia	F/P^+^ patient: no biological or clinical response
									+5 doses to 2 patients with biological (>30 d) and clinical response: magnitude and duration of response decreased Rebound at 6–8 wks
									BM 4 wks post-Tx: no effect on eosinophilia and cellularity

Stein et al. (19) MEPO NCT00266565	Open-label 10 mg/kg (max 750) IV 3× 4 wks	19–57	19^c^	Systemic HES1 imatinib-resistant F/P^+^ patient Run-in: reduction of BL therapy to achieve twofold increase of EOS or EOS > 0.75 G/L	Evaluation of impact on immune function (Table 4)	Reduced in 18 patients 4 wks after 3rd dose Responders: 26-fold reduction, duration 3 mo in 10/14 assessed	Not assessed	Not assessed	3 cohorts on the basis of % reduction in BL HES Tx during study: A 0%, B 25%, and C 50% Rebound HE only observed in cohort C
									F/P^+^ patient responded to MEPO, EOS counts normal until wk28
									The only non-responder had highest IL-13 levels in PHA-stimulated-PBMC supernatants

Rothenberg et al. (42) MEPO NCT00086658 MHE100185	RDB PC (OCS tapering) 750 mg IV 12× 4 wks	Adults, mean 48.1	85	Systemic F/P^−^ OCS-responsive HES (Chusid’s definition) Run-in: stabilized with PDN monotherapy (20–60 mg/d), EOS < 1 G/L	PDN dose ≤10 mg for ≥8 wks: 84 versus 43%	Eos < 0.6 G/L for ≥8 wks: 95 versus 45%If BL PDN > 30 mg: 100 versus 8%	Not assessed	Not assessed	difference in primary EP achievement btw MEPO and PLAC more pronounced in patients requiring > 30 mg PDN at BL: 77 versus 8%
								Ability to taper down OCS while maintaining disease stability considered surrogate for clinical response	
									daily PDN dose decreased from roughly 30 mg at BL (mean in all patients) to 6.2 mg with MEPO and 21.8 mg with PLAC
									tapered off OCS until study completion: 47 versus 5%

Roufosse et al. (20) MEPO NCT00097370 MHE100901	Open-label (extension of MHE100185) 750 mg IV 5 yrs 4 wks (stage 1), then variable (stages 2 and 3)	18–75, median 50	78	Eligible if participated in MHE100185 (completed or received at least 2 doses of study Tx)	Long-term safety:safety confirmed, No recurrent drug-related AEs/SAEs leading to Tx interruption	Mean EOS < 0.5 G/L in all but 1 patient in stage 2(1 non-responder, EOS count unchanged)	Not assessed	5 withdrawals due to lack of efficacy on HES symptoms/signs54 continued until end of study PDN ≤ 10 mg end of study: 83% PDN-free during study: >50%	Study design with 3 stages: (1) tapering of background Tx to minimal effective dose, assessment of EOS response, (2) determination of optimal dosing interval in responders (re-dosing if EOS > 0.6 G/L or disease manifestations present), (3) dosing at fixed intervals Dosing interval end stage 2: >12 wks in 50% patients; median 12.8 wks; range 21–37 wks

**Eosinophilic granulomatosis with polyangiitis**									

Kim et al. (69) MEPO NCT00527566	Open-label (OCS tapering) 750 mg IV 4× (28-wk FU) 4 wks	28–62, mean 45	7	OCS-depend EGPA, PDN ≥ 10 mg (ANCA status unknown) At baseline: mean daily PDN 12.9 mg; IS (MTX) 3/7, disease stable, mean EOS 3.4%	PDN dose reduction: mean 4.6 mg 4 wks after 4th dose (64% reduction)	Reduced mean EOS to 0.8% at end of active Tx	Not assessed (no change in Fe NO)	Decreased exacerbation rate during active Tx (compared with washout and FU) Decreased ACQ Unchanged FEV1	Prolonged PDN dose reduction:mean 5 mg 12 wks after 4th dose Return to 15.7 mg 28 wks (7 mo) after last dose

Moosig et al. (70) MEPO NCT00716651	Open-label (OCS tapering) 750 mg IV 9× 4 wks	43–78, mean 62	10	Relapsing/refractory EGPA despite PDN ≥ 12.5 mg and IS (ANCA status unknown) At inclusion: median daily PDN 19 mg, no IS, BVAS ≥ 3, active organ involvement	BVAS 0 with daily PDN<7.5 mg: achieved by 8/10 patients	Mean 0.026 at end of active Tx phase (for the 9/10 patients that completed)	Not assessed	No exacerbations during active Tx period No change in FEV1 at end of active Tx	PDN dose reduction:From median daily PDN dose 19 mg at BL to 4 mg at time of 9th dose ^d^Follow-up study with MTX 0.3 mg/kg as maintenance Tx (9 patients): 3 had prolonged remission (median FU 22 mo), 6 relapsed (delay 4.5 mo to >2 yrs)

Wechsler et al. (64) MEPO NCT02020889 MEA115921	RDB PC 300 mg IV 8× 4 wks	Mean 48.5	136	Daily PDN dose required to control EGPA 7.5–50 mg History: asthma + EOS > 1 G/L + 2 criteria typical of EGPA19% ANCA^+^, 75% required IS	*Remission (BVAS 0 with PDN* ≤ *4* *mg*) – Accrued REM ≥ 24 wks: 28 versus 3% – REM at wks 36 + 48: 32 versus 3%	Significant decrease in active Tx arm only (not detailed)	Not assessed	Twofold lower relapse rate in MEPO arm (1.14 versus 2.27)	PDN dose reduction: Higher proportion of patients at PDN dose ≤4 mg for the last 4 wks (44 versus 7%) Higher proportion of patients able to stop PDN during trial (18 versus 3%)
								Higher proportion of patients experienced REM in MEPO arm (53 versus 19%)	
									Treatment benefit significant only in patients with BL blood EOS > 0.15 G/L: 33 versus 0% had REM ≥ 24 wks

*^a^For placebo-controlled trials, the reported findings concern statistically significant (unless mentioned otherwise) differences observed between the active treatment arm and the placebo arm; the response rate to active treatment arm is reported first, followed by response to placebo. Several non-significant differences judged worth underlining are mentioned as well*.

*^b^One patient in this study had EoE and was also included in Ref. (47) [see Stein et al. (47), Table 2]; not included in this table*.

*^c^Three patients with HES were already reported in Ref. (41) [see Garrett et al. (41), Table 3]; 6 patients in this cohort with EoE are not included in this table*.

*^d^Extended post-Tx follow-up was reported for 9 of the 10 patients in a separate publication (71)*.

*abd, abdominal; ACQ, asthma control questionnaire; (S)AE, (serious) adverse event; ANCA, antineutrophil cytoplasmic antibody; BL, baseline; BM, bone marrow; btw, between; BVAS, Birmingham vasculitis activity score; (O)CS, (oral) corticosteroid; d1, day 1; ECP, eosinophil cationic protein; EGPA, eosinophilic granulomatosis with polyangiitis; EOS, eosinophil; EP, endpoint; FeNO, fractional exhaled nitric oxide; FEV1, forced expiratory volume in 1 s; F/P, FIP1L1–PDGFRA; FU, follow-up; HES, hypereosinophilic syndrome; IL-5, interleukin-5; IS, immunosuppressor; IV, intravenous; MEPO, mepolizumab; mo, month; MTX, methotrexate; Nbr, number; OCS, oral corticosteroids; PBMC, peripheral blood mononuclear cells; PDN, prednisone; PE, primary endpoint; PHA, phytohemagglutinin; PLAC, placebo; RDB PC, randomized double-blind placebo-controlled; REM, remission; RESLI, reslizumab; SAE, serious adverse events; SC, subcutaneous; Tx, treatment; wk, week; yr, year*.

### Idiopathic HES

Patients with idiopathic HES may present with single (organ-restricted) or multiple (complex) organ involvement. Single-organ disorders comprise most commonly chronic eosinophilic pneumonia, gastroenteritis, and dermatitis. In complex HES, two or more organs/systems are affected (skin, lungs, digestive tract, heart and/or blood vessels, central and/or peripheral nervous system, and coagulation), and certain complications may be life-threatening. Reduction of blood and tissue eosinophilia is critical to prevent and reverse organ damage, and together with control of disease complications, represents the major goal of treatment (72). Most patients with HES respond to systemic CS, but many patients require second-line CS-sparing agents, none of which are fully safe and/or effective. The most commonly used agents are hydroxyurea and interferon-alpha (IFN-α), followed by other cytotoxic or immunosuppressive drugs, and by stem cell transplantation for the most refractory cases. Because of their ability to deplete eosinophils rapidly and specifically, IL-5 pathway-targeting antibodies represent attractive therapeutic options for these disorders. Among the three available antibodies, only mepolizumab has been proven effective in HES in the setting of a randomized placebo-controlled clinical trial. Past and ongoing studies with reslizumab and benralizumab are summarized in Tables 3 and 5.

**Table 4:** Biological effects of IL-5(R) targeted therapy other than eosinophil depletion in diseases other than asthma.^a^

	Eosinophils	T cells	Mast cells	Remodeling	Serum/mediators	Tissue/mediators
**Eosinophilic esophagitis**						

Stein et al. (47) MEPO Esophagus	No change in CCR3 expression by blood EOS		Twofold decrease Eso MC (in 3 out of 4 patients)	Decreased epithelial hyperplasia (in 3 out of 4 patients)		

Straumann et al. (48) MEPO Esophagus	Unchanged expression of IL-5Rα by blood EOS	No effect on Eso CD3 T cells	No effect on Eso MC (tryptase^+^)	Reduced epithelial TGFβ1 + tenascin C (effect delayed, most marked 4 wks after 4th dose)	Decreased ECP + EDN Increased eotaxin	Reduced EDN^+^ cells and extracellular EDN deposition>60% reduction eotaxin-1,2,3 and IL-5 positive cells in esophagus Unchanged Eso epithelial expression of eot-3 and TNFα

Otani et al. (59) MEPO EsophagusResponders*: <*15 EsoEOS/hpf (40%)*	Reduced Eso EOS degranulation and clusters Reduced IL-9^+^ EOS in Eso epithelium		Decreased Eso MC in 77% patients after 3rd infusion Responders*: threefold decrease Eso MC, sixfold decrease EOS/MC couplets, correlation btw MC and EOS counts			Responders*: Reduced epithelial IL-9^+^ cells (NS reduction non-EOS IL-9^+^ cells) *Ccl: eos induce/sustain MC through IL-9 production, or MC are directly targeted by anti-IL-5*

**Chronic rhinosinusitis with nasal polyposis**						

Gevaert et al. (50) RESLI Nasal polyps					Reduced sIL-5Rα and ECP Eotaxin unchanged	Nasal secretions: Reduced sIL-5Rα, ECP Reduced IL-5 only in responders Eotaxin unchanged

Gevaert et al. (51) MEPO Nasal polyps					Reduced sIL-5Rα and ECP	Nasal secretions: Reduced sIL-5Rα, IL-6, IL-1β (*impact on tissue neutrophils not investigated*) ECP, IL-5, IgE unchanged

**Hypereosinophilic syndrome**						

Plotz et al. (40) MEPO Dermatitis		Modest reduction in skin T cells (CD4 and CD8) Reduced production IL-4/5/13 by PHA-stimulated PBMC (in 2 out of 3 patients)			Reduced ECP, IL-5, TARC, eotaxin	

Kim et al. (26) RESLI HES—EGID	Unchanged survival *in vitro*, in medium ± IL-5 *Indirectly indicates unchanged IL-5R*α *expr*.	Unchanged % of IL-5, IL-3, GM-CSF, IFN-γ-expressing PMA/iono-stimulated T cells			Unchanged IL-2, IL-3, IL-8, IL-15, GM-CSF, IFNγ, TNFy	
					IL-5 decreased 2–3 d post-Tx (3 patients), then increased at 1 mo (5/6 patients)	

Stein et al. (19) MEPO HES	Increased expression of IL-5Rα (unchanged CCR3) by blood EOS Reduced shape change in response to eotaxin-1,2,3	Increased % IL-5 expressing PMA/iono-stimulated T cells in 8 (CD4) and 7 (CD8) out of 12 patients Unchanged % IL-4/IL-13/IFNγ/TNFα^+^ T cells			Increased IL-5 in 60% subjects: high MW, precipitated by protein A/G (complexed with immunoglobulin)	Increased production IL-13 by PHA-stimulated PBMC in 13 out of 20 patients (no change in IL-4, IL-5, IFNγ, IL-10, AND GM-CSF)

Rothenberg et al. (42) MEPO HES					Decreased EDN	

*^a^This table summarizes posttreatment findings (active treatment). See Tables 2 and 3 for details on the clinical trials during which these observations were made, except for Otani et al. (59) [this was a follow-up biomarker study performed with biopsy samples obtained in the clinical trial published by Assa’ad et al. (18)]*.

*btw, between; ccl, conclusion; d, day; ECP, eosinophil cationic protein; EDN, eosinophil-derived neurotoxin; EGID, eosinophilic gastrointestinal disease; EOS, eosinophil; Eot, eotaxin; Eso, esophageal epithelial; FC, flow cytometry; GM-CSF, granulocyte macrophage colony stimulating factor; gp, group; hpf, high-power field; IL-5, interleukin-5; iono, ionomycin; MC, mast cell; MEPO, mepolizumab; MPO, myeloperoxidase; MW, molecular weight; NS, non-significant; PBMC, peripheral blood mononuclear cells; PHA, phytohemagglutinin; PMA, phorbol 12-myristate 13-acetate; RESLI, reslizumab; TARC, thymus- and activation-regulated chemokine; TNF, tumor necrosis factor; Tx, treatment; wk, week*.

**Table 5:** Ongoing and planned clinical trials in eosinophilic disorders other than asthma using IL-5 targeted therapy.

Drug	Dosing	Design	Primary endpoint Secondary endpoints	Comments	Clinicaltrials.gov identifier
**Eosinophilic esophagitis (pediatric)**					

Reslizumab	1–3 mg/kg IV every 4 wks	Open-label	Long-term safety and efficacy	Study completed, not published112/190 enrolled subjects completed the study; 28/78 withdrawals due to lack of efficacy	NCT00635089

**Chronic rhinosinusitis with nasal polyposis**					

Mepolizumab	100 mg SC every 4 wks (13 doses)	Phase 3, RDB PC Severe bilateral NP Add-on maintenance therapy	Endoscopic nasal polyp score Nasal obstruction VAS score	6-mo extension study for half of the patients	NCT03085797

Reslizumab	3 mg/kg IV every 4 wks (6 doses)	Phase 3, RDB PC	Change in NP CT score (imaging)		NCT02799446

Benralizumab	Not available Tx period 24 wks	Phase 2, RDB PC	Change in NP score (endoscopy)		NCT02772419 Japan

**Hypereosinophilic syndrome**					

Mepolizumab MHE104317	Initially 750 mg IV (250 mg vials), variable interval Currently, 100 mg vials; sponsor recommendation: 300 mg IV every 4 wks	Compassionate use program for life-threatening HES with documented failure of/intolerance to ≥3 standard Tx Long-term access program for patients who participated in a previous HES study	Support provision of mepolizumab until commercially available for HES	Subjects ≥12 yrs Regular evaluation of risk:benefit ratio to support continued treatment More than 200 patients enrolled	NCT00244686

Mepolizumab MID200622	300 mg SC every 4 weeks (9 doses)	Phase 3, RDB PC History of 2 flares 12 mo before enrollment, EOS ≥ 1 G/L on stable Tx at inclusion	Proportion of patients who experience a flare	Adolescents ≥12 yrs eligible Followed by 20-wk open-label study 205203	NCT02836496

Benralizumab	30 mg SC every 4 wks (total study duration 1 yr)	Phase 2, RDB PC (3 mo) followed by active Tx in all Refractory HES (EOS > 1 G/L despite Tx)	50% reduction blood EOS on stable HES background Tx at wk12	Study completed, results awaited	NCT02130882

**Eosinophilic granulomatosis with polyangiitis**					

Mepolizumab MEA116841	300 mg SC every 4 wks	Open-label	Systemic CS use Adverse events	Long-term access program for MEA115921 participants who require ≥5 mg PDN	NCT03298061

Benralizumab	30 mg SC, 5 injections over 32 wks	Open-label	Safety Change in OCS dose and exacerbation rate		NCT03010436

Reslizumab	3 mg/kg IV every 4 wks (7 doses)	Open-label	Safety CS-sparing effect		NCT02947945

*(O)CS, (oral) corticosteroid; EOS, eosinophil; IL-5, interleukin-5; IV, intravenous; mo, month; NP, nasal polyposis; OCS, oral corticosteroids; PDN, prednisone; RDB PC, randomized double-blind placebo-controlled; SC, subcutaneous; Tx, treatment; VAS, visual analog scale; wk, week; yr, year*.

Efficacy of mepolizumab in patients with HES was first tested in two small open-label pilot studies. In the first (40), three patients with OCS-resistant eosinophilic dermatitis and blood eosinophil counts above 1.5 G/L received IV mepolizumab infusions (750 mg), resulting in normalization of blood eosinophilia within 1 day, and rapid improvement of cutaneous manifestations. In contrast to the initial asthma trials, tissue eosinophils were practically undetectable in posttreatment hematoxylin–eosin-stained biopsies (although ECP staining was reduced but not abolished). In the second study (41), 3 monthly mepolizumab infusions were administered to three patients with complex HES, after an initial run-in period during which their maintenance therapy was tapered to a level such that blood eosinophil levels increased at least twofold or rose above 0.75 G/L. Treatment resulted in profound and prolonged eosinophil depletion, symptom improvement, reduced nasal polyp volume, and increased FEV1.

These promising results encouraged the conception of the first randomized double-blind placebo-controlled trial ever conducted in subjects with F/P-negative HES to date (MHE100185) (42). Before randomization, patients had to be clinically stable and have eosinophil counts below 1 G/L with OCS monotherapy (daily prednisone-equivalent dose at least 20 mg, but no more than 60 mg). One week after study-treatment commenced, prednisone was tapered according to a predefined algorithm based on clinical manifestations and blood eosinophilia. Patients in the active treatment arm were significantly more likely to achieve the primary endpoint (prednisone dose 10 mg or less for a period of at least eight consecutive weeks), and the difference with placebo was particularly marked in those who needed more than 30 mg at baseline. Other secondary/exploratory CS-sparing endpoints establishing superiority of mepolizumab over placebo included a significant reduction of the mean daily prednisone dose at the end of the study (mepolizumab 6.2 mg and placebo 21.8 mg), and a higher proportion of patients able to taper off OCS treatment completely until the end of the trial (47 versus 5%). Although blood eosinophilia is much higher in this disease than in asthma, EoE, and CRSwNP (defining criteria >1.5 G/L), mepolizumab-treated patients were more likely to maintain counts below 0.6 G/L than placebo-treated patients, despite the fact that the OCS dose was lower in the former group during treatment. Although effects on HES-related complications were not evaluated in this study (patients were stabilized at baseline), clinical deterioration requiring a major escalation in treatment (i.e., treatment failure) was experienced by 21% of mepolizumab-treated versus 69% placebo-treated subjects, and time to treatment failure was significantly shorter in the placebo arm. Most (84%) mepolizumab-treated patients completed the trial, whereas only 36% of placebo-treated patients did, the main reason for withdrawal being lack of efficacy.

Patients who participated in the MHE100185 trial were eligible for enrollment in an open-label extension study (MHE100901) designed to assess the long-term safety and optimal dosing interval of 750 mg IV mepolizumab in HES (20). This study included three stages (see Table 3) and lasted 5 years. During stage 1, mepolizumab was administered monthly, and background HES therapy was tapered off, or down to the minimal dose required for disease control. During stage 2, mepolizumab infusions were spaced, and administered only when blood eosinophilia (>0.6 G/L) and/or clinical manifestations recurred. More than half the patients were CS-free by week 48, and the proportion off CS remained constant until the end of the study (63%). The median average daily prednisone dose during the entire study was 1.8 mg, and only three patients required addition of other immunosuppressive medications for HES control. The optimal dosing interval between infusions (median 12.8 weeks) was relatively reproducible for each individual over the prolonged observation period, with half of the patients requiring re-treatment after more than 12 weeks. This study was not designed to evaluate efficacy, but did provide indirect confirmation that mepolizumab benefits patients with HES, since only 6 of the 78 enrolled patients withdrew because of lack of efficacy (persistent blood hypereosinophilia in 1, and HES-related symptoms in 5), and 54 were still receiving treatment when the study ended more than 5 years later. Practically speaking, for a meaningful proportion of CS-dependent patients with long-standing HES, this trial resulted in replacement of daily OCS absorption by a visit to the hospital every 3 months for a 30-min mepolizumab infusion. After study termination, patients were given the opportunity to continue treatment on a compassionate use basis and many are still receiving mepolizumab at time of writing.

The compassionate use program (MHE104317) is also open to subjects aged 12 or more with life-threatening HES and documented failure to at least three standard therapies (e.g., CS, hydroxyurea, IFN-α, and imatinib mesylate). Patient and disease characteristics, exposure to mepolizumab, and safety data are being collected in this cohort (73). Case reports showing efficacy of mepolizumab for severe treatment-refractory idiopathic HES have been published, including one patient with recurrent arterial thrombosis in extremities (74) and another with eosinophilic myocarditis and pericardial effusion (75). In children, toxicity of classical HES therapies is a major concern, contrasting with the favorable safety profile of mepolizumab, explaining that some children with severe HES enter this program as soon as they are 12 (76).

Despite the observed efficacy of mepolizumab in HES, this agent has not yet been approved in this indication. One reason is that clinical efficacy has not yet been formally proven (see A Brief Historical Perspective on the Development of IL-5 Targeted Therapy for Human Diseases). An ongoing clinical trial in HES has derived some useful lessons from asthma studies, choosing exacerbation rate reduction as primary endpoint (Table 5), and should provide more insight on how mepolizumab impacts disease manifestations. Although there is some concern that efficacy may be lower than in the previous placebo-controlled trial because of reduced dosing (300 mg SC rather than 750 mg IV) in patients whose eosinophil levels can be very high, data from the compassionate use program suggest that many patients continue to do well when the IV dose is lowered.

### Lymphocytic-Variant HES (L-HES)

In L-HES, hypereosinophilia is driven by a clonal population of activated T cells that over-produce IL-5 in vivo (77). In the majority of cases, these cells can be distinguished in peripheral blood on the basis of their abnormal surface phenotype (CD3^−^CD4^+^) by lymphocyte phenotyping. Patients with L-HES have elevated serum levels of thymus-and- activation-regulated chemokine (TARC), a chemokine that presumably reflects *in vivo* production of Th2 cytokines (78).

Although anti-IL-5 efficacy has not been evaluated specifically in patients with L-HES, a biomarker sub-study was conducted during the MHE100185 trial to identify these patients at baseline, and to compare their treatment response to that of patients with a normal T cell profile (79). Among patients with appropriate testing, 13/63 were classified as L-HES on the basis of T cell phenotyping (predominantly CD3^−^CD4^+^), and 33/81 had elevated serum TARC levels. In the active-treatment arm (monthly mepolizumab 750 mg infusions), patients with phenotypically abnormal T cells were as likely as those with a normal T cell profile to achieve the CS-sparing endpoints, as were patients with elevated versus normal serum TARC values. However, the ability to maintain eosinophil counts below 0.6 G/L throughout the trial was significantly lower in patients with T cell-driven HES: (1) 71% patients with an abnormal phenotype, versus 100% patients with a normal phenotype and (2) 73% patients with serum TARC > 1,000 pg/mL versus 100% patients with TARC. During the MHE100901 open-label study that immediately followed this trial, treatment response was compared between these patient sub-populations as well (personal observation). No statistically significant differences were observed in terms of long-term CS-sparing. However, the interval between mepolizumab infusions (750 mg) in patients with CD3^−^CD4^+^ L-HES was roughly half that of subjects with phenotypically normal T cells, and mean eosinophil levels 4 weeks after each infusion during stage 2 were significantly higher, although still within normal limits. It remains unclear whether these subtle differences in treatment response between patients with L-HES and non-T cell driven HES have clinically relevant consequences, as neither trial was designed to explore the efficacy of mepolizumab on disease manifestations. It is noteworthy that a complete clinical and biological (eosinophils <0.5 G/L) response to mepolizumab (dosing not detailed) was observed in four of five patients with L-HES in the largest L-HES cohort published to date (80).

Altogether, these observations suggest that in some patients with T cell-driven HES, higher and/or more frequent dosing of anti-IL-5 may be required to neutralize the large amounts of IL-5 produced *in vivo*. Patients with L-HES will certainly be enrolled in the ongoing clinical trial evaluating mepolizumab in HES, and their response to the 300 mg SC dosing regimen will be evaluated and compared with that of patients with idiopathic HES in the setting of a biomarker sub-study. Notwithstanding, provided dosing is sufficient, anti-IL-5 treatment does allow for CS tapering in many L-HES patients and represents an extremely well tolerated alternative to the high-dose CS maintenance treatment they often require. Until T cell targeted treatment directed against pathogenic cells has been developed for this HES variant, anti-IL-5 antibodies fulfill a strong unmet need.

### EGPA (Formerly Churg–Strauss Syndrome)

Eosinophilic granulomatosis with polyangiitis is a complex disorder combining peripheral blood hypereosinophilia, severe asthma, eosinophil-rich and granulomatous inflammation in lungs and other organs, and small/medium-vessel necrotizing vasculitis (81, 82). Classically, disease develops in three consecutive stages: (1) progressive adult-onset asthma, often associated with chronic rhinosinusitis, (2) peripheral blood hypereosinophilia with eosinophilic infiltrates in lungs and possibly other organs, and (3) vasculitis. The disease is heterogeneous, with underlying pathogenic mechanisms that presumably differ in patient sub-groups. Patients with positive ANCA serology for example are more likely to develop purpura, glomerulonephritis, pulmonary hemorrhage, and mononeuritis multiplex than ANCA-negative subjects. An operational approach to diagnosis requires presence of asthma, sinusitis and/or rhinitis, pathological confirmation of vasculitis or clinical surrogates highly evocative of vasculitis in at least two organs, and blood eosinophilia above 1.5 G/L. The vasculitic component often responds durably to immunosuppressive drugs such as cyclophosphamide, rituximab, and azathioprine, but the majority of patients remain OCS-dependent because of asthma exacerbations and chronic rhinosinusitis. Methotrexate or azathioprine may be required as maintenance therapy for CS-sparing purposes.

Although it is unclear whether eosinophils contribute directly to the vasculitic features of EGPA, they do infiltrate lungs and account for dyspnea in stage 2 disease. At this stage, ANCA-negative EGPA is often indistinguishable from chronic eosinophilic pneumonia (single-organ HES) or idiopathic HES (83, 84) (when organs other than lungs are affected as well). Because HES patients with lung involvement responded well to mepolizumab in the MHE100185 trial, and because anti-IL-5 is efficacious in severe eosinophilic asthma (which is a key feature of EGPA), it is not surprising that IL-5-targeted treatment has been tested in EGPA.

The first attempt to treat EGPA with mepolizumab was published in 2010 as a case report (85). A patient with multiorgan ANCA-negative EGPA resistant to high-dose prednisolone and various immunosuppressive and cytotoxic agents experienced a clear-cut response to monthly mepolizumab infusions (750 mg) with regression of blood eosinophilia, lung infiltrates and asthma, and normalization of pulmonary function tests. An attempt to increase the interval between doses led to an exacerbation, which was controlled when monthly infusions were resumed.

This observation was closely followed by two pilot single-center open-label studies in the United States (69) and in Germany (70). The first study enrolled OCS-dependent patients whose disease was controlled with stable background therapy at inclusion. During the active treatment period, 4 monthly mepolizumab infusions (750 mg) were administered, and the mean dose of prednisone required to maintain disease control was significantly lowered, from 12.9 mg at baseline to 4.6 mg 4 weeks after the fourth dose. The CS-sparing effect was prolonged an additional 2 months, but the dose had to be increased thereafter. The exacerbation rate was significantly lower during the active treatment phase compared with the washout and follow-up periods. The second study included patients with more severe EGPA, whose disease was active at baseline (BVAS 3 or more) despite more potent background therapy (daily OCS dose at least 12.5 mg combined with an immunosuppressive agent). Besides lung and sinus involvement, other organs were affected in most subjects (heart, digestive tract, and peripheral nervous system). Nine monthly mepolizumab infusions (750 mg) were administered after tapering off immunosuppressant(s), and 8/10 patients experienced a clinical remission (BVAS 0) with a prednisone dose below 7.5 mg. A significant reduction of the median prednisone dose was observed (19 mg at baseline versus 4 mg on the day of the ninth infusion), and no disease exacerbations were observed during treatment. Thus, during 9 months, disease activity was abrogated despite cessation of maintenance immunosuppressive therapy and decreased prednisone dosing in the majority of these patients with severe EGPA.

These very encouraging pilot studies led the European Commission to grant orphan designation to mepolizumab for the treatment of EGPA in 2013, and a large-scale placebo-controlled clinical trial was designed to evaluate efficacy in patients with relapsing or refractory EGPA requiring daily prednisone intake (between 7.5 and 50 mg) with or without concomitant immunosuppressive agent(s) to stabilize disease (MEA115921) (64). Treatment (mepolizumab 300 mg or placebo SC) was administered every 4 weeks for 52 weeks, and investigators were blinded to eosinophil counts to guarantee double blinding. The two primary efficacy endpoints related to remission, which was defined as a BVAS of 0 with a PDN dose of 4 mg or less. There was a statistically significant difference in favor of mepolizumab-treated patients, who were more likely to experience an accrued remission of 24 weeks or more (28 versus 3%), and to be in remission at weeks 36 and 48 (32 versus 3%). Like in asthma, treatment benefit on the relapse rate was significant only in patients with blood eosinophil counts above 0.15 G/L at inclusion. There was a twofold lower relapse rate in the mepolizumab group (1.14 versus 2.27), although a higher proportion of these patients had tapered the prednisone dose to 4 mg or less at the end of the trial (44 versus 7% in the placebo arm), and some even completely discontinued OCS treatment during the trial (18 versus 3%). Globally, IL-5 targeted therapy maintained disease control despite OCS tapering in roughly half of subjects with EGPA. A supplemental Biologics License Application seeking approval for mepolizumab as add-on therapy to OCS for EGPA has been submitted to the FDA.

Compared with patients with HES enrolled in the MHE100185 trial, the response rate in EGPA is disappointing. This may be due to the reduced dosing, and/or key involvement of additional IL-5-independent pathogenic mechanisms in this complex disease. The dose chosen for the EGPA trial (300 mg SC), although higher than for eosinophilic asthma (100 mg SC), was one-third of that used for HES (750 mg IV). Patients with EGPA often have markedly increased blood eosinophilia, similar to patients with HES. Serum TARC levels are often elevated, and CRTH2-postive cells have been detected in nasal tissue, suggesting pathogenic involvement of Th2 cells. Furthermore, the first patient with EGPA who responded to monthly high-dose mepolizumab experienced a relapse when the dosing interval was increased. It is therefore conceivable that, like for L-HES, certain patients with EGPA may require higher dosing to neutralize higher endogenous IL-5 production. The contribution of pathogenic complexity to variable treatment responses in EGPA is difficult to assess on the basis of data collected during clinical trials. Neither histological findings before inclusion showing vasculitis nor ANCA status were reported in the two pilot studies. In the placebo-controlled trial, baseline characteristics indicate that both patients with vasculitic disease (ANCA-positivity, alveolar hemorrhage, palpable purpura) and patients with stage 2 EGPA (asthma, blood eosinophilia at least 1 G/L, pulmonary infiltrates, sinonasal abnormalities) were enrolled, but the small size of clinical sub-groups precluded statistical comparisons in response rates.

Likewise, no conclusions can be formally drawn regarding the effects of mepolizumab on the different components of EGPA: asthma, sinonasal disease, and vasculitis. In the recent placebo-controlled trial, benefit of active treatment was slightly greater on relapses that were defined as worsening asthma or rhinosinusitis, although relapses considered as both vasculitic and asthma/sinonasal were also reduced. In the pilot study conducted by the German group, there were no flares during the active treatment phase although these patients had been tapered off immunosuppressive therapy and OCS dosing was reduced, whereas after treatment cessation, six out of nine patients with extended follow-up relapsed, including two patients who developed progressive neuropathy and alveolar hemorrhage respectively (71), suggesting that vasculitic manifestations of EGPA may have been controlled during treatment with anti-IL-5. Alternatively, these findings may reflect natural disease course, with protracted relapses occurring in patients who had been tapered off azathioprine for the purpose of the clinical trial.

The ongoing NIH-funded biomarker sub-study conducted on biological material obtained from a subset of patients enrolled in MEA115921 may identify disease characteristics, subsets, and biomarkers that are predictive for treatment response and disease exacerbations.

## Anti-IL-5 Treatment in Other Inflammatory Disorders Associated with but Not Exclusively Driven by Hypereosinophilia

Several complex inflammatory diseases associated with symptomatic hypereosinophilia have been shown to benefit from treatment with mepolizumab, the only anti-IL-5 antibody available for such indications through the compassionate use program, or insurance company approval for use of commercialized 100 mg vials.

### Chronic Inflammatory Disorders with Hypereosinophilia

In some chronic inflammatory and/or indolent hematological diseases with accompanying eosinophilia, (partial) symptomatic improvement may be obtained by targeting eosinophils, even if the underlying condition does not warrant (or respond to) specific therapy. Given the excellent safety profile of mepolizumab, this holds especially true if the toxicity of disease-modifying treatment exceeds the anticipated benefit.

Elderly patients with bullous pemphigoid, for example, may not tolerate immunosuppressive therapy at doses required to stabilize disease. Eosinophils are present in skin lesions and are often increased in peripheral blood, and recent studies indicate that they contribute to pathogenesis (86). Targeting eosinophils may therefore represent a future therapeutic alternative for this disease (87). A 3-month phase 2 placebo-controlled trial evaluating the efficacy of monthly infusions of mepolizumab (750 mg) in adult patients with active bullous pemphigoid has recently been completed and results should be available shortly. Drug reaction with eosinophilia and systemic symptoms (DRESS), a potentially life-threatening eosinophil-mediated inflammatory disorder triggered by an inappropriate immune response to certain therapeutic agents [see Musette and Janela in this topic (88)], may also be an interesting indication for short-term anti-IL-5 treatment in patients with particularly CS-refractory or recurrent manifestations (89).

Eosinophils may also contribute to symptoms and complications in certain indolent hematological disorders, such as mastocytosis and cutaneous T cell lymphoma, that need not necessarily be treated aggressively. One patient with unrecognized cutaneous mastocytosis associated with hypereosinophilia responded to prolonged treatment with mepolizumab (750 mg IV), before the correct diagnosis was made more than 10 years after initial presentation (90). Eosinophil counts normalized and she experienced symptomatic improvement (pruritus, erythematous eruptions, and chronic cough) with repeated mepolizumab infusions. It remains unclear whether symptoms were due to eosinophils themselves, or to mast cells that may have been directly (if they expressed the IL-5R) or indirectly [abolished production of an eosinophil-derived mediator involved in mast cell growth or activation, like IL-9 (91)] targeted by treatment. Although anti-IL-5(R) may indeed offer some relief in chronic hematological conditions like this, it should be kept in mind that the role played by eosinophils on natural disease course remains elusive, and that eosinophil depletion may jeopardize negative regulatory pathways acting on clonal cells.

### Combined Therapy with Other Monoclonal Antibodies

Mepolizumab has successfully been administered concomitantly with other monoclonal antibodies to treat complex immune-mediated diseases driven by more than one cell type and/or mediator.

Mepolizumab improved disease course in a patient with atypical hemolytic uremic syndrome (aHUS), who responded poorly to eculizumab (anti-C5a) alone (92). Monthly mepolizumab injections were initiated because of associated blood and tissue (colon) eosinophilia, resulting in normalization of eosinophil and platelet counts, increased ADAMTS13 activity, and regression of digestive and neurological manifestations. This observation suggests that hypereosinophilia and aHUS may enhance one another, with C3a and C5a enhancing eosinophil activation, and conversely, eosinophil-induced endothelial damage exacerbating thrombotic microangiopathy. The authors conclude that complement dyscrasias with an eosinophilic component may benefit from anti-IL-5 therapy.

Monthly administration of mepolizumab significantly improved disease course in a wheelchair-bound CS- and oxygen-dependent patient with severe allergic bronchopulmonary aspergillosis who responded only partially to omalizumab (93). She was able to discontinue OCS and oxygen, and to resume activities of daily living for the first time in years after addition of mepolizumab. These interesting case reports provide insight on new pathogenic roles played by eosinophils in complex disorders.

### A Word of Caution

Although the often dramatic effect on eosinophilia and excellent safety profile of anti-IL-5 treatment understandably rouse enthusiasm, the priority should be given to compounds that specifically target disease-inducing defects when available, or to other less expensive options if their efficacy and toxicity are satisfactory. For example, lymphomatoid papulosis associated with symptomatic hypereosinophilia was shown to (transiently and partially) respond to mepolizumab in one patient (94). However, this condition may be observed in subjects with F/P^+^ CEL, and should be treated with imatinib mesylate when this is the case. Another group reported a young woman with ulcerative colitis and marked blood hypereosinophilia who responded to combined mepolizumab and infliximab (95). She had initially failed to respond clinically to mepolizumab, although blood eosinophilia regressed. Repeat biopsies showed active colitis with cryptitis, infliximab was administered, and together, these monoclonals resulted in clinical and biological remission. However, no attempt to discontinue mepolizumab was reported, to determine whether infliximab alone would have sufficed to control disease and resolve hypereosinophilia.

It is also important to judge whether eosinophils are indeed contributing to organ damage or dysfunction at all (i.e., “is there a hypereosinophilic *syndrome*”?), and not to administer eosinophil-targeted therapy if this is not the case. For example, mepolizumab was administered to a subject with blood and (sub)cutaneous eosinophilia in the setting of angiolymphoid hyperplasia with eosinophilia (ALHE, or epitheloid hemangioma) (96). Hypereosinophilia and local pruritus disappeared, but the subcutaneous nodule regressed only slightly. Pathogenesis of ALHE is not well delineated, but many cell types are involved, and it is unlikely that eosinophils represent the predominant driving force.

In summary, treatment with anti-IL-5 antibodies is expensive and justified only when other therapies fail and/or have a negative impact on health or quality of life, and there is reasonable evidence that the role played by eosinophils in organ damage or dysfunction is significant. Isolated reports showing efficacy in conditions for which other safe treatment options exist should not encourage physicians to squander health care resources and resort to anti-IL-5 treatment whenever eosinophils are present.

## Predictors of Response/Resistance to IL-5 Targeted Therapy

Little is known about disease characteristics that are predictive of a response to anti-IL-5 treatment in patients with hypereosinophilic conditions.

### Clinical Presentation

Clinical trials evaluating anti-IL-5 in patients with HES are few and have given no clear indication on specific disease manifestations whose presence may predict treatment response. In the MHE100185 trial, patients with active cutaneous involvement at enrollment had a slightly lower response rate to mepolizumab (69%) than those with active respiratory, gastrointestinal, or cardiac manifestations (90–100%) (42). However, this finding may reflect disease severity, as 72% of patients requiring more than 30 mg prednisone at baseline had active cutaneous involvement (versus only 37% of those requiring 30 mg or less). In the same trial, patients with L-HES receiving active treatment were less likely to maintain eosinophil counts below 0.6 G/L than patients with normal T cells (79) and required more frequent dosing in the follow-up dosing-interval study MHE100901. Their requirement for higher dosing is likely related to higher endogenous production of IL-5 by the dysregulated T cells that drive disease.

The type of eosinophil-mediated complications more likely to regress with treatment have not been studied either, as clinical outcome has not been a major endpoint so far. In L-HES, part of the clinical manifestations may actually be related to T cell over-produced cytokines other than IL-5, such as IL-2, IL-4, IL-13, and TNF-α.

### Eosinophil Counts

Patients with eosinophilic asthma who were most likely to respond to IL-5 targeted treatment in the placebo-controlled trials that led to FDA approval were those with higher baseline blood eosinophil counts. For mepolizumab, a relationship was observed between exacerbation rate reduction and baseline eosinophilia (97), and for reslizumab and benralizumab, clinical and functional responses were observed in patients with baseline eosinophilia at or above 0.4 and 0.3 G/L, respectively (13). Likewise, in the EGPA mepolizumab-versus-placebo trial, a reduction in the exacerbation rate was observed only in patients whose baseline eosinophilia was 0.15 G/L or more (64).

In patients with other HES, the relationship between eosinophil counts before treatment and treatment response has not been evaluated, because in most studies, baseline eosinophilia was controlled with maintenance OCS therapy. In one open-label study where maintenance therapy was tapered down so that eosinophil counts were increasing at the time mepolizumab was initiated, the extent of the decrease in eosinophil counts in response to treatment was most marked in the patient with the highest baseline eosinophilia (>1.5 G/L) (19).

### Serum Biomarkers of Eosinophil Activation

Eosinophil expression of membrane IL-5Rα may decrease, and soluble IL-5 receptor (sIL-5Rα) levels may increase, in tissue and body fluids from patients with eosinophilic inflammation, as a result of alternative splicing and/or shedding (36, 50). Incubation of eosinophils *in vitro* in presence of IL-5, IL-3, and GM-CSF results in decreased IL-5Rα expression, and a correlation has been shown between serum IL-5 and sIL-5Rα levels in subjects with HES (36). It has been hypothesized that sIL-5Rα may bind IL-5 and trap anti-IL-5 antibodies; it may also be recognized by the anti-IL-5R antibody benralizumab and prevent it from binding to target cells. The impact of increased pretreatment serum sIL-5Rα levels on efficacy of anti-IL-5 treatment has only been assessed in one study evaluating reslizumab in CRSwNP, showing no relationship (50).

### IL-5 Production

Both eosinophils and T cells have the capacity to produce IL-5 and release it in serum. Baseline serum IL-5 levels were measured in patients with HES that participated in several trials with anti-IL-5, and no correlation with treatment response was observed (25, 41, 42). Background therapy at baseline probably lowered the serum IL-5 level, making it impossible to determine the utility of this marker. Moreover, IL-5 levels in serum may reflect imperfectly the degree of IL-5 production at sites of inflammation and in lymphoid tissue. IL-5 was measured in nasal secretions in mepolizumab-treated subjects with nasal polyposis to address this issue, initially demonstrating that levels above 40 pg/mL were predictive of a clinical response (50), although this was not confirmed in a subsequent larger-scale study conducted by the same group (51). Demonstration of local IL-5 expression in biopsies from eosinophil-rich tissues would represent more convincing evidence of the role played by IL-5 in inflammation. Very few biopsy studies have been conducted in the setting of anti-IL-5 clinical trials for hypereosinophilic conditions. The ongoing NIH-funded biomarker sub-study on patients who participated in the mepolizumab/EGPA trial may provide insight on the association between local Th2 inflammation and a specific treatment response profile.

Another means of quantifying the Th2 response *ex vivo* is to investigate cytokine production by PBMC. This was undertaken by one group who evaluated the efficacy of open-label mepolizumab in subjects with HES (19). Interestingly, the single patient who did not respond to treatment was the one whose PBMC secreted the highest amounts of IL-13 in culture supernatants, suggesting that patients with marked *in vivo* Th2 activation may not respond as well to treatment, like patients with L-HES. Evaluation of the cytokine profile of *in vitro*-stimulated PBMC as a means of detecting Th2-driven disease in patients with HES is not routine or standardized, however, and requires expertise.

### Serum Biomarkers of Th2-Driven Disease

Measurement of TARC in serum may be a more reliable means of detecting Th2-driven inflammation in tissues, where TARC is produced by resident cells. Elevated levels have been observed in subjects with T cell lymphoma, and atopic dermatitis amongst others. In the MHE100185 trial, HES patients with serum TARC levels above 1,000 pg/mL were less likely to maintain blood eosinophil counts below 0.6 G/L than those with low TARC levels, among those receiving active treatment (78). So far, this is the only standardized biomarker shown to be associated with a suboptimal treatment response. In this era of precision medicine, measurement of serum TARC may help identify patients less likely to respond to anti-IL-5, and/or more likely to require higher or more frequent dosing. The predictive value of baseline and peak serum TARC levels for response to mepolizumab will be evaluated prospectively in a biomarker sub-study within the ongoing MID200622 clinical trial.

Like TARC, eotaxins could potentially reflect local Th2 inflammation. In one study, plasma eotaxin-3 levels were not predictive of a response to mepolizumab (19).

## Indirect Effects of IL-5 Targeting on Cell Types Other than Eosinophils

Besides eosinophils, cell types expressing the IL-5R in humans and thereby predictably targeted by anti-IL-5(R) treatment include basophils, and certain mast cell subsets. In addition, eosinophils entertain numerous interactions with their environment (98), and it is legitimate to explore indirect effects of IL-5-targeted treatment, resulting from eosinophil depletion, on other cell types *in vivo*. Overall, no changes in blood leukocyte counts (besides eosinophils) have been observed in the numerous trials with anti-IL-5 for asthma and eosinophilic disorders. Pre- versus posttreatment lymphocyte sub-populations were enumerated in mepolizumab-treated subjects, showing no significant changes in blood CD3, CD4, CD8, CD19 (B cell), CD16/56 (NK cell), or γ/δ cell counts (19, 40, 99). Benralizumab depletes basophils in addition to eosinophils, and the mild and transient decrease in other white blood cells (lymphocytes, monocytes, and neutrophils) initially observed with rapid intravenous administration of this compound (28) has not been reported subsequently with subcutaneous dosing.

Effects of IL-5-targeted treatment on numbers and functional properties of several relevant cell types and on inflammatory mediators in patients with hypereosinophilic conditions other than asthma are detailed in Table 4.

## Safety of Therapeutic Antibodies Targeting IL-5 and its Receptor

### Adverse Events (AE) in Clinical Trials

The most obvious means of evaluating safety is to compare AE recorded in patients receiving active treatment to patients receiving placebo in the setting of clinical trials. The safety data from severe eosinophilic asthma trials, and in less common diseases such as EoE, HES and EGPA, has been reviewed in detail and will not be developed herein (44, 100–102). No major concerns have been raised so far, after more than 10 years of data collection with anti-IL-5; the occurrence of AE has been overall similar to placebo, both in number and nature (most commonly headache, nasopharyngitis, and upper respiratory tract infection). Some of the reported AE are related to tapering of background treatment (20) (e.g., symptoms/signs of adrenal insufficiency, unmasking of unrelated CS-responsive inflammatory conditions such as rheumatoid arthritis), and/or specific disease components responding less well to eosinophil-targeted therapy (e.g., persistent airway hyperreactivity in patients with severe asthma). None of the clinical trials conducted with mepolizumab or reslizumab have raised any major concerns with regard to anaphylaxis, or infusion/injection site reactions. The acute infusion-related AEs that were initially observed with intravenous benralizumab were easily resolved by increasing the duration of the infusion (28) and have no longer been an issue with subcutaneous dosing. Although benralizumab induces eosinophil cell death extremely rapidly, there is no evidence that degranulation products are released massively at treatment initiation. Indeed, serum levels of eosinophil-derived granule proteins actually decrease early after treatment initiation in asthmatic patients (31). No specific clinical AE have repeatedly been considered by investigators as drug-related with any of these antibodies.

The limitation of clinical trials for obtaining information on safety is their short duration, with the exception of the MHE100901 study during which no major safety concerns were raised in more than 50 patients receiving mepolizumab for HES, despite the fact that mean exposure in those receiving more than one infusion was almost 5 years (20). Cough, fatigue, headache, upper respiratory tract infection, and sinusitis were the most common AE in roughly one-third of subjects each. Patients who participated in the pivotal placebo-controlled trials evaluating efficacy of mepolizumab in asthma (101) and EGPA were given the opportunity to receive open-label treatment in the setting of long-term access programs that have not raised major safety concerns although safety data collection has spanned prolonged periods.

### Consequences of Prolonged Eosinophil Depletion

Now that anti-IL-5 antibodies have been commercialized and widespread use in patients with severe asthma is anticipated, phase 4 safety data may provide some insight on effects of prolonged eosinophil depletion on human biology in terms of host defense against infections, cancer, and possibly other immune and non-immune mechanisms. Indeed, besides causing damage in inflammatory disorders, eosinophils contribute to protective immune responses directed against selected parasites, and there is accumulating evidence that they are involved in defense against viral, bacterial, and fungal pathogens (5). Experimental findings indicate a possible role in anti-tumor immunity, although their increased presence in cancer does not necessarily correlate with favorable outcome. Eosinophils also contribute to maintenance of a healthy immune response (e.g., crucial for the survival of long-lived plasma cells), and it is believed they represent critical regulators of local immunity and remodeling/repair in both health and disease (103). Their presence in various tissues in healthy subjects [i.e., homeostatic eosinophils (9)] is increasingly being explored at the functional level, and experimental evidence indicates that they regulate an array of biological functions including control of glucose homeostasis, protection against obesity, regulation of mammary gland development, and preparation of the uterus for pregnancy. It is therefore logical to question whether prolonged therapeutic eosinophil depletion may impact some of these processes.

This concern was addressed by a group of experts in eosinophil biology, who reviewed clinical and experimental observations made in humans (case reports on eosinopenic subjects) and murine strains devoid of eosinophils (104). Eosinopenia was not associated with increased susceptibility to infections, cancer or any other major abnormalities in homeostasis impacting global health. They concluded that, although eosinophils do contribute to many immune and physiological processes, they appear to be dispensable, thanks to the existence of other eosinophil-independent pathways and are not critical for maintenance of homeostasis in mammals.

Safety data from clinical trials is in agreement with this interesting review, namely, with regard to susceptibility to infections. The rare occurrence of herpes zoster infections that were considered serious in patients receiving mepolizumab (23) (open-label extension studies) or benralizumab (105) may eventually justify vaccination before treatment initiation. Helminthic infections are uncommon in industrialized countries in the Northern hemisphere where anti-IL-5(R) antibodies have been administered so far. Patients who travel regularly or who reside in countries where helminths are endemic should be followed carefully now that anti-IL-5 treatment is widely available for severe asthma. There is no data suggesting increased susceptibility to opportunistic infections.

Regarding tumor surveillance, the majority of neoplasms that have developed during clinical trials with anti-IL-5 are those that are most common in the general population (100) (e.g., basal cell carcinoma, prostate cancer, and squamous cell carcinoma). Their incidence appears not to be meaningfully higher than expected on the basis of the SEER database, taking into account the close observation of patients during clinical trials. The only exception is T cell lymphoma. Indeed, the incidence of lymphoma in mepolizumab-treated patients with HES was higher than expected in a control population (20). However, it is well known that HES may precede diagnosis of T cell lymphoma, either because occult lymphoma is not detected at the time a patient presents with paraneoplastic hypereosinophilia, or because the eosinophil-driving disease itself progresses to T cell lymphoma (e.g., L-HES). Thus, occurrence of lymphoma in an anti-IL-5-treated patient with HES is more likely to reflect progression of underlying disease (or its unmasking, following reduction of background treatment) than a treatment-effect on clonal T cells. This is supported both by the fact that the two patients who developed T cell lymphoma in the MHE100901 study had markedly elevated serum TARC levels (a marker of underlying T cell disease) before initiation of treatment with mepolizumab (20), and by the fact that lymphoma has not occurred in the many anti-IL-5(R)-treated patients with asthma, a disease that has no inherent relationship with the development of this malignancy (100).

As for homeostatic functions, it appears that eosinophils in certain compartments are less dependent on IL-5 for their persistence than inflammatory eosinophils. For example, homeostatic lung eosinophils that have been shown to suppress Th2-driven allergic airway responses do not express the IL-5R (9). Likewise, resident duodenal eosinophils do not consistently express IL-5Rα, and they are not affected in mepolizumab-treated patients with EoE (106), which together with unchanged mast cells and T cells, suggest preserved local host defense.

Finally, it should be noted that the effects of anti-IL-5 antibodies on eosinophil depletion in tissues is partial (often roughly 50%), so eosinophils likely continue to play their physiological roles. The more pronounced effect of benralizumab on tissue eosinophils may affect certain functions more profoundly. Long-term safety data is being collected in patients with severe asthma.

### Effects of IL-5 Targeting on Eosinophil Activation

Treatment with antibodies against soluble ligands is known to potentially impact receptor-bearing cells at a functional level. For example, omalizumab decreases the expression level of the IgE receptor by mast cells, and this is believed to contribute to clinical efficacy. IL-5 is known to prime eosinophils *in vivo* for trafficking and activation in response to other inflammatory mediators, so neutralizing this cytokine may render the eosinophils that remain less reactive to other stimulatory pathways and less prone to perpetuate inflammation. One study has shown that eosinophils isolated from blood of mepolizumab-treated patients were indeed less responsive *in vitro* to eotaxins in terms of eosinophil shape change compared with pretreatment eosinophils (19) (Table 4). However, another group recently showed that in mepolizumab-treated asthmatic subjects subjected to allergen challenge, airway eosinophils express an activated phenotype similar to that observed before treatment, suggesting that this eosinophil subset does conserve its functional properties in these conditions despite anti-IL-5 treatment (107).

Increased membrane expression of the IL-5α receptor chain by eosinophils has been observed after treatment with anti-IL-5 (19), raising concern about a potentially enhanced response to endogenous IL-5 after treatment cessation, with rebound hypereosinophilia (i.e., posttreatment eosinophilia higher than baseline level), and/or eosinophil degranulation. Rebound eosinophilia and disease manifestations have not been observed with mepolizumab, despite the fact that it has been evaluated extensively in asthma and several other eosinophilic conditions, including studies with prolonged follow-up (48, 51). By contrast, rebound hypereosinophilia and severe symptom exacerbations occurred between 60 and 90 days after treatment in six of six patients with HES or eosinophilic gastroenteritis whose eosinophil levels initially decreased in response to a single dose of 1 mg/kg reslizumab (26). A similar phenomenon was observed in asthma and nasal polyposis trials, but was clearly related to lower dosing regimens (50, 108). It has been suggested that at low molar antibody/cytokine ratios, biologically active cytokines may be released from their complexed form and/or may retain their capacity to bind to and activate their receptor (109, 110). This phenomenon may contribute to rebound eosinophilia and/or disease worsening with anti-IL-5 treatment. In this line, rebound hypereosinophilia was observed only in HES patients treated with reslizumab (1 mg/kg), who received a substantially lower dose of antibody than those treated with mepolizumab (10 mg/kg). With the currently approved dose of reslizumab (3 mg/kg), no major concerns about rebound eosinophilia have been raised in diseases with low baseline eosinophil counts, such as asthma or EoE.

### Potential Consequences of Reduced Dosing Regimens

Over time, the dosing regimen of mepolizumab has progressively decreased in clinical trials, passing from 750 mg IV in the first asthma and HES studies, to 100 mg SC in asthma and 300 mg SC in HES. The consequences of administering low-dose anti-IL-5 treatment are an increasing subject of concern (111, 112), for several reasons besides potential rebound hypereosinophilia. These include reduced efficacy, and the possible development of an immune complex-mediated inflammatory response in the setting of excess antigen. Indeed, as the antibody:antigen ratio decreases, there is an increased likelihood that both Fab sites of each antibody will be occupied, leading to the formation of immune complexes that are more likely to precipitate and activate complement (113). One group has recently reported the case of a prednisone-dependent asthmatic patient whose airway disease worsened dramatically while treated with mepolizumab 100 mg daily (114). The authors hypothesized that local immune complex deposition, activation of complement, and recruitment of immune cells may have caused uncontrolled inflammation. Increased sputum IL-5 levels (bound to antibody) and eosinophilia were observed as lung function deteriorated, suggesting that mepolizumab prolonged the half-life of biologically active IL-5, resulting in maturation of eosinophil progenitors in the airways. This observation further underscores the likelihood that OCS-dependent asthmatic subjects have a more severe disease (115), with higher needs in terms of anti-IL-5 dosing, to reach the airways and effectively neutralize the target cytokine locally in addition to the intravascular compartment (112).

## Future Perspectives and Closing Remarks

Specific targeting of eosinophils has become possible and is now implemented in daily clinical practice, with antibodies directed against IL-5 and its receptor. For eosinophil-driven chronic inflammatory conditions, targeting IL-5-dependent pathways enables a much-needed shift from global immunosuppression to precision medicine, with improved safety and tolerance. How this translates into clinical improvement of complex diseases depends on how central eosinophils are in pathogenesis. For several HES variants, the benefit has been outstanding, whereas for diseases driven by several mediators and cell types with various phenotypes and endotypes, such as severe asthma and EoE, IL-5 targeting has variable efficacy. Improved understanding of pathogenic disease mechanisms on one hand, and more profound eosinophil depletion in tissues in ongoing and future clinical trials with benralizumab on the other hand, will help gain insight on the extent and nature of eosinophil involvement in various complex inflammatory states and determine which disorders will benefit most from treatment with anti-IL-5(R).

With increasing use of these antibodies for hypereosinophilic conditions in clinical practice, optimal dosing regimens should be the focus of future attention. Indeed, dosing should be tailored to patient needs in a more personalized approach to treatment than that implemented in clinical trials, taking into account both efficacy and tolerance. Ideally, similar to other immune-modulatory agents, the minimal clinically efficacious dose should be tailored to each patient/condition, as the potential negative impact of long-term eosinophil depletion on the quality of global immune responses remains unknown. The amount of antibody administered at once, and/or the interval between doses may be titrated to this end. The likelihood that sustained IL-5 targeting eventually reverses natural disease course is low, as confirmed in asthmatic patients whose eosinophilia and symptoms recurred after discontinuation of mepolizumab (116) and in most patients with EGPA who were shifted to maintenance treatment with methotrexate after 9 monthly infusions (71). In patients with idiopathic HES, disease may exceptionally regress over time, either spontaneously, or as the result of prior immunosuppressive/cytotoxic treatment, which may explain that prolonged remission after cessation of anti-IL-5 has been reported in a few cases (20, 40, 71). It may be worthwhile to carefully taper selected patients off anti-IL-5 treatment in the future, to explore this possibility. On the other hand, patients in whom endogenous IL-5 production is particularly intense (e.g., L-HES and subsets of patients with idiopathic HES and EGPA) should be offered the possibility of increasing the dose before concluding that treatment is ineffective or not justified economically. This concept is implemented in omalizumab-treated asthmatic patients whose dose is adapted to serum IgE levels. Unfortunately, there are as of yet no validated biomarkers reflecting the level of endogenous IL-5 production that could be used to guide the choice of dosing of anti-IL-5 in patients with marked hypereosinophilia, although serum TARC levels appear promising in this regard (79). Changes in approved dosing regimens should be evaluated in the setting of carefully monitored clinical studies. Post-marketing authorization data is awaited for mepolizumab in the phase 4 “multinational single-arm observational study to evaluate the real-world effectiveness and pattern of use of mepolizumab in patients with severe eosinophilic asthma” (100). Clinicians and patients will likely be tempted to increase between-dose intervals when disease is stabilized, and such modifications require close follow-up as they may result in disease worsening, due to decreases in the antibody:antigen ratio and/or release of biologically active IL-5.

Special attention should also be paid to the consequences of tapering background therapy in patients with chronic eosinophilic disorders whose condition improves significantly after initiation of treatment with anti-IL-5. Indeed, variable degrees of adrenal insufficiency develop over time after prolonged systemic and even topical CS use (117), and its under-estimation may result in serious complications during CS tapering. In a recent open-label extension study with mepolizumab in asthmatic patients, the maintenance dose of OCS was higher when tapering was left to the clinicians’ discretion, than when done in the setting of placebo-controlled clinical trial constraints (i.e., following a predefined algorithm to reach the lowest possible dose), indicating that clinicians naturally take the risks and discomforts associated with OCS withdrawal into account (101). Furthermore, a subset of asthmatic patients successfully treated with low-dose (100 mg) anti-IL-5 and tapered off OCS may progress to EGPA, as previously reported for other agents like leukotriene antagonists and omalizumab (115). All of these eventualities mandate close monitoring.

Finally, with the increasing availability of compounds targeting other mediators and cells involved in chronic inflammatory diseases associated with eosinophilia, optimal treatment strategies may involve combination therapy. Identification and increased availability of reliable biomarkers predicting treatment response will help design efficacious and minimally toxic tailored treatment regimens for patients with these complex disorders in great need of well-tolerated therapeutic options.

## Author Contributions

FR designed the scope and wrote this manuscript.

## Conflict of Interest Statement

FR has served as consultant for GlaxoSmithKline for clinical trial design with mepolizumab for hypereosinophilic syndromes.
